# Global Trends in Integrating Machine Learning (ML) with Model-Informed Drug Development (MIDD): A Bibliometric and Systematic Review (2015–2025)

**DOI:** 10.3390/pharmaceutics18050542

**Published:** 2026-04-28

**Authors:** Doni Dermawan, Samir Chtita, Nasser Alotaiq

**Affiliations:** 1 Department of Applied Biotechnology, Faculty of Chemistry, Warsaw University of Technology, 00-661 Warsaw, Poland; doni.dermawan.stud@pw.edu.pl; 2 Laboratory of Analytical and Molecular Chemistry, Faculty of Sciences Ben M’Sik, Hassan II University of Casablanca, Casablanca 20670, Morocco; samirchtita@gmail.com; 3 Health Sciences Research Center (HSRC), Imam Mohammad Ibn Saud Islamic University (IMSIU), Riyadh 13317, Saudi Arabia

**Keywords:** artificial intelligence, bibliometric analysis, deep learning, machine learning, model-informed drug development, pharmacometrics, physiologically based pharmacokinetic modeling, precision pharmacology, quantitative systems pharmacology

## Abstract

**Background/Objectives**: The integration of machine learning (ML) within model-informed drug development (MIDD) represents a rapidly evolving paradigm in pharmacometrics, enabling improved prediction, optimization, and regulatory decision-making across drug development pipelines. However, the extent to which ML methods are explicitly integrated into regulatory decision-making remains limited and unevenly characterized. This study aims to systematically map the ML-MIDD scholarly landscape, identify core sources and contributors, assess thematic evolution, and summarize key methodological advancements through an integrative bibliometric and systematic review. **Methods**: A comprehensive literature search was conducted across Web of Science, Scopus, and PubMed (2015–2025), followed by metadata harmonization and deduplication. Bibliometric analysis was performed using Bibliometrix, VOSviewer, and PRISMA guidelines to characterize publication trends, collaboration patterns, thematic structures, and representative methodological contributions. **Results**: A total of 770 records were initially retrieved, with Scopus contributing the largest share (*n* = 343; 44.5%), followed by Web of Science (*n* = 322; 41.8%) and PubMed (*n* = 105; 13.6%). After deduplication, 607 unique publications remained (78.8% of total), and 560 were included in the final systematic review (97.6% of full texts). Publications spanned 269 sources, with core journals accounting for 28% of output. The United States led in volume (*n* = 665; 20.8%) and international collaboration (16.47%). Thematic evolution revealed transitions from foundational PK/PD methods (2016–2018) to applied ML-driven precision pharmacology (2022–2025). **Conclusions**: Emerging methods included deep learning, reinforcement learning, and hybrid mechanistic–ML models. ML-MIDD is a rapidly maturing interdisciplinary field, evidenced by expanding methodological diversity and increasing use of ML-enabled components within regulatory-relevant modeling workflows, rather than formal regulatory endorsement of ML-MIDD as a standalone methodology, indicating growing translational relevance but continued need for validation and regulatory clarity.

## 1. Introduction

Drug development remains extraordinarily costly and risky. For example, it has been estimated that bringing a new drug to market now takes over a decade and costs on the order of hundreds of millions of dollars (mean ~$515.8 million), while roughly 90% of candidates fail during clinical development [1,2]. Model-informed drug development (MIDD) is a quantitative pharmacology paradigm that uses integrated pharmacokinetic/pharmacodynamic (PK/PD) and exposure-response models to inform decisions throughout the drug development lifecycle [3,4]. By leveraging techniques such as population PK/PD, physiologically based PK (PBPK), and model-based meta-analysis, MIDD aims to improve the efficiency of development and reduce cost and risk [5,6]. In recent years, MIDD has become a foundational pillar of modern drug research and development, as Dunn and van der Graaf note, MIDD has emerged as a foundational pillar of modern drug development and is now routinely embedded in regulatory and industry decision-making, providing tools to optimize study design, dose selection, and benefit-risk assessment [7]. Importantly, the core goal of MIDD is individualized (precision) pharmacology. A study emphasizes that MIDD methods play a critical role in ensuring the development of efficacious and safe individualized therapies [8]. Furthermore, MIDD integrates diverse mechanistic models so that pharmacological understanding from drug exposure to effect can directly support precise dosing and trial design decisions [9].

Complementing this trend, virtual bioequivalence (VBE) and in silico modeling have emerged as vital extensions of MIDD, leveraging PBPK modeling to predict pharmacokinetic profiles and reduce reliance on extensive clinical trials. These advancements not only enhance drug development efficiency but also align with evolving FDA and EMA regulatory frameworks, underscoring MIDD’s transformation into a regulatory cornerstone for virtual trials and informed decision-making in complex formulations [10]. For example, the FDA’s MIDD Paired Meeting Program (2023–2027) explicitly notes that MIDD approaches can “improve clinical trial efficiency, increase the probability of regulatory success, and optimize drug dosing/therapeutic individualization” [11].

Artificial intelligence (AI) refers to computational methods designed to perform tasks that typically require human intelligence, including pattern recognition, reasoning, decision-making, and prediction [12]. Machine learning (ML) represents a core subfield of AI focused specifically on algorithms that learn patterns from data and improve performance over time without being explicitly programmed. Thus, ML is one of the primary operational engines through which AI capabilities are realized. Supervised learning, unsupervised learning, and reinforcement learning are major ML families that enable prediction, clustering, and sequential decision processes, respectively [13,14]. These principles form the basis for many AI-powered applications in healthcare and drug development. Concurrently, ML and other AI methods have surged in biomedical research, offering powerful tools for data integration and prediction. In healthcare broadly, ML is transforming the analysis of real-world data (RWD): for example, a recent systematic review notes that ML and big data analytics are rapidly transforming health care, particularly disease prediction, management, and personalized care [15].

In healthcare broadly, ML is rapidly transforming the analysis of real-world data (RWD). For example, a systematic review of ML applied to RWD (2014–2024) identified 57 studies (total *n* > 150,000 patients) and noted that ML and big data analytics are “rapidly transforming health care”, especially in disease prediction, management, and personalized care [15]. However, that review also found many (60%) of studies faced challenges in data quality, model interpretability, and generalizability. Within drug development, ML is increasingly paired with traditional pharmacometrics. A recent review reports that integrating ML into pharmacometric modeling “has the potential to optimize dosing strategies, inform clinical trial designs, and enhance robustness of quantitative assessments of drug efficacy and safety” [8]. For instance, Zhu et al. developed an XGBoost model that “bridges big data and pharmacometrics” by combining covariates from different population PK models [16]. These examples illustrate how ML, the data-driven subset of AI, can augment MIDD by enabling more flexible pattern recognition, handling high-dimensional patient and biological datasets, and complementing mechanistic models to enhance predictive power and decision-making. Industry trends underscore this convergence: for example, the global AI in pharmaceutical market was about $1.94 billion in 2025 and is projected to reach ~$16.5 billion by 2034 (compound annual growth rates/CAGR ~27%) [17], reflecting the rapid growth of AI integration in drug R&D. These examples illustrate how ML can augment MIDD. ML handles high-dimensional patient or biological data that complements mechanistic models, enhancing predictive performance while preserving interpretability [18,19].

The convergence of MIDD and ML represents a paradigm shift toward precision pharmacology, where data-driven and mechanistic approaches are combined to tailor therapy. By uniting classical PK/PD modeling with flexible ML algorithms, researchers can more precisely optimize dosing and individualize treatment [20,21]. The AI/ML integration in MIDD has immense promise for personalized therapy design, but also underscores the need for methodological rigor. A study highlights the lack of standardized evaluation metrics and regulatory guidance for AI-enabled MIDD methods [8]. AI applications in drug development provide a foundation for future research to optimize and integrate AI-based approaches in this field. Similarly, another study highlights ML’s bridging role by integrating outputs from multiple PK models; ML can harness “big data and pharmacometrics” together, paving the way for innovative modeling frameworks [16]. These insights suggest a research landscape moving decisively toward hybrid model development, a synthesis of mechanistic and machine-learned components, to achieve individualized drug therapy.

Despite these advances, a comprehensive mapping of the ML-MIDD field remains lacking. Most prior studies have been case-based or methodological in nature, and systematic analyses are limited. For instance, Mao et al. conducted a systematic review of AI-MIDD methods, highlighting current limitations and research needs but without a bibliometric perspective [8]. This study, therefore, seeks to map the global evolution, key contributors, and research topics in ML-MIDD between 2015 and 2025. Using bibliometric and systematic review approaches, it aims to identify publication trends, influential authors and institutions, and emerging methodological themes, thereby illuminating how the integration of ML and MIDD is shaping the future of precision pharmacology.

## 2. Materials and Methods

### 2.1. Data Sources and Search Strategy

This bibliometric and systematic review was designed to systematically map the global research landscape on the integration of ML with MIDD from 2015 to 2025. To ensure comprehensive coverage, three major bibliographic databases were queried: Web of Science (Clarivate Analytics, Philadelphia, PA, USA), Scopus (Elsevier, Amsterdam, The Netherlands), and PubMed (U.S. National Library of Medicine, Bethesda, MD, USA). These databases were selected for their broad coverage of biomedical and pharmaceutical sciences and their complementary indexing features. Web of Science and Scopus were prioritized for their robust bibliometric metadata and citation tracking capabilities, while PubMed was included for its extensive biomedical scope and use of controlled vocabulary (Medical Subject Headings, MeSH). The search strategy was developed to maximize both recall and precision, focusing on the intersection of ML-related terms with established MIDD methodologies, such as PBPK simulations. Boolean operators and database-specific field tags were adapted for each platform to ensure optimal retrieval. The generalized search query was:

(“machine learning” OR “artificial intelligence” OR “deep learning” OR “neural network”) AND (“model-informed drug development” OR “PBPK” OR “QSP” OR “PopPK” OR “pharmacokinetic modeling” OR “dose optimization”) AND (“drug development” OR “pharmacometrics” OR “precision pharmacology”)

Where applicable, controlled vocabularies were incorporated, using MeSH in PubMed and index keywords in Scopus, to enhance search accuracy. The temporal filter was applied to include studies published between 1 January 2015, and 30 September 2025, corresponding to the emergence and rapid expansion of ML and AI applications in pharmacometrics and quantitative systems pharmacology (QSP). To ensure a structured data mining process, all retrieved search results underwent a multi-step pipeline consisting of (i) raw data export from each database, (ii) initial automated filtering, (iii) format standardization, and (iv) centralized consolidation for downstream screening and deduplication. Each database export included full metadata records containing titles, abstracts, author information, author keywords, indexed terms, citation counts, DOIs, and journal details. All search results were exported in standardized bibliographic formats (BibTeX, Comma-Separated Values/CSV, or Research Information Systems/RIS) for further cleaning, deduplication, and bibliometric analysis. Metadata extracted included publication title, authors, affiliations, keywords, abstracts, citation counts, journal names, and publication years. The complete database-specific search strings and parameters are provided in Supplementary Data S1.

### 2.2. Inclusion and Exclusion Criteria

Studies were considered eligible if they met the following inclusion criteria:

- Topical relevance: Articles must explicitly address the application, integration, or methodological development of ML, AI, or deep learning (DL) approaches within the context of MIDD, including PBPK, population PK (PopPK), or QSP.
- Pharmaceutical or pharmacometric focus: Studies had to pertain directly to drug discovery, development, or clinical pharmacology, particularly in dose optimization, exposure–response modeling, or precision pharmacology.
- Methodological or applied content: Articles must describe either original methodological advances, applications, or frameworks employing ML with MIDD techniques rather than purely conceptual or speculative discussions.
- Timeframe: Publications had to appear between 1 January 2015 and 30 September 2025, representing the established cut-off for literature inclusion used across the Abstract, Introduction, and main text. This timeframe corresponds to the decade in which ML-driven pharmacometric research expanded substantially, and the full details of this search window are also documented in Supplementary Data S1.

Exclusion criteria were defined to ensure dataset precision and reproducibility. Studies were excluded if they:

- Lacked accessible bibliographic metadata necessary for bibliometric analysis.
- Focused solely on AI/ML in general biomedical or healthcare settings without explicit linkage to MIDD, PBPK, PopPK, or QSP.
- Reported only non-pharmacometric modeling (e.g., image analysis, clinical decision support, or Electronic Health Record/EHR prediction) unrelated to drug development.
- Articles were published in non-English languages.

This selection framework ensured that the final dataset reflected original, data-driven research integrating ML and MIDD methodologies, suitable for bibliometric, network-based evaluation, and systematic review.

### 2.3. Data Extraction and Processing

A standardized bibliometric data extraction framework was implemented to ensure consistency, interoperability, and reproducibility across all retrieved records. For each publication, comprehensive metadata were extracted, including the title, abstract, author keywords, indexed keywords, journal name, publication year, and citation metrics. Authorship information was collected in detail, encompassing the full list of contributing authors, their institutional affiliations, and countries of origin, thereby enabling subsequent analyses of collaboration networks at individual, institutional, and national levels. Citation-related variables, such as total citation counts and average citations per year, were retrieved to assess publication impact and research productivity. As part of the structured data mining workflow, all metadata fields were first programmatically parsed and normalized to ensure consistent labeling, field completeness, and compatibility across databases. Missing fields (e.g., absent DOIs or incomplete author lists) were flagged automatically for later inspection. Abstracts, titles, and keywords were text-processed to remove non-standard characters, unify capitalization, and harmonize terminology prior to keyword co-occurrence and thematic analyses.

To support cross-platform compatibility, all bibliographic data were exported in multiple standardized formats, including BibTeX, RIS, and CSV. These interoperable formats facilitated downstream processing and integration with diverse analytical tools. All bibliometric operations were performed using the Bibliometrix R-package version 4.1.2 and its Biblioshiny graphical interface version 5.0 (University of Naples Federico II, Naples, Italy) [22], which provided a transparent, reproducible environment for data handling and analysis. The extracted dataset served as the foundation for descriptive analyses (e.g., annual publication trends, top journals, and leading authors), relational analyses (e.g., co-authorship and institutional collaboration networks), and thematic mapping (e.g., keyword co-occurrence and conceptual structure). This structured approach ensured that both quantitative and qualitative dimensions of the ML-MIDD research landscape were systematically captured and analyzed. The final output of this data mining stage was a unified, structured dataset containing harmonized metadata fields ready for deduplication, relational mapping, and thematic analysis.

### 2.4. Data Harmonization and Deduplication Workflow

All bibliographic records retrieved from Web of Science, Scopus, and PubMed were systematically harmonized, merged, and deduplicated using a custom R-based workflow developed by the authors. The workflow was implemented in R version 4.5.2 (R Foundation for Statistical Computing, Vienna, Austria) and utilized multiple specialized packages to ensure reproducible data integration, standardization, and visualization [23]. The data mining workflow followed a stepwise structure consisting of: (i) format unification (conversion of RIS/BibTeX/CSV files into a common dataframe structure), (ii) field standardization (ensuring consistent variable names and data types), (iii) hierarchical, rule-based deduplication across sources using a multi-criteria matching sequence (DOI matching followed by exact-title, normalized-title, and author-string similarity matching), (iv) text normalization (lowercasing, removal of accents, punctuation harmonization), and (v) final manual verification of ambiguous duplicates. This custom algorithm was selected because metadata discrepancies across Web of Science, Scopus, and PubMed, particularly missing DOIs, inconsistent punctuation, and differences in author formatting, reduce the accuracy of default automated tools such as Bibliometrix. The hierarchical matching strategy substantially improves duplicate detection and cross-database integration, especially for records with incomplete or non-standardized metadata.

While the full R script implementing this workflow is restricted by funding-related data management policies and cannot be publicly released, the analytical framework is provided in the form of pseudocode in Supplementary Data S2 to ensure methodological transparency. The bibliometrix package version 4.1.2 (University of Naples Federico II, Naples, Italy) [22] served as the core engine for bibliographic data import, conversion, and integration, using its convert2df() and mergeDbSources() functions to standardize metadata fields (e.g., title, authors, DOI, year, and source journal) and to detect and remove duplicates across sources automatically. The openxlsx package version 4.2.8.1 (OpenXML Developer Community, various contributors, Worldwide) [24] facilitated the structured export of the merged and deduplicated dataset into Microsoft Excel (.xlsx) and CSV formats for downstream analyses. Data cleaning and normalization were performed using the stringi package version 1.8.7 (Gagolewski Research Group, Melbourne, Australia) [25], which standardized textual metadata (e.g., titles and author names) through lowercasing, accent removal, and punctuation stripping to generate consistent identifiers even for records lacking DOIs. The grid (R Core Team, Vienna, Austria) version 3.6.2 and cowplot packages version 1.2.0 (Wilke Lab, University of Texas at Austin, Austin, TX, USA) [26] were used to manage complex figure layouts and combine multiple visual components into publication-ready graphics, while ggplot2 (Wickham et al., Posit PBC, Boston, MA, USA) [27] provided the foundation for high-quality bar charts depicting database-specific publication counts. The VennDiagram package version 1.7.3 (Chen & Boutros Lab, University of Toronto, Toronto, ON, Canada) [28] was employed to generate a three-set Venn diagram that visualized overlaps among Web of Science, Scopus, and PubMed datasets, highlighting shared and unique publication counts following deduplication. The workflow produced two complementary visualizations: (1) A triple Venn diagram illustrating database overlap; and (2) A bar chart showing the distribution of unique records per database. These plots were merged into a unified high-resolution figure titled “Overlap of Publications Across Databases (2015–2025)”, annotated with the total number of unique records obtained after deduplication. The harmonized dataset generated through this pipeline provided a robust foundation for subsequent bibliometric analyses, including co-authorship mapping, keyword co-occurrence analysis, and thematic evolution.

### 2.5. Bibliometric and Network Analysis

The analytical workflow followed established bibliometric procedures [22] and was explicitly aligned with the research questions (RQs) guiding this study. Only RQs relevant to bibliometric objectives were included in the present section, while the full list of RQs is reported in Supplementary Data S3. To address RQ1 (current state of publication) and RQ2 (publication trends), descriptive statistics were applied to summarize document types, source types, subject areas, languages, and contributor characteristics (authors, institutions, countries, and journals). Annual publication counts, citation trajectories, and CAGR were computed to characterize the temporal evolution of ML-MIDD research. These quantitative trends were interpreted in the context of key scientific and regulatory milestones, including the expansion of MIDD frameworks, regulatory guidance from the FDA/EMA, and the increasing adoption of AI/ML tools in pharmacometrics and quantitative systems pharmacology.

RQ3 and RQ4 (productive and influential contributors) were examined by ranking authors, institutions, and countries using both productivity metrics (publication counts) and impact indicators (total citations, h-index, g-index, and m-index). Core journals were identified through Bradford’s Law, revealing a high concentration of influential work in pharmacometrics, computational modeling, and drug development outlets, supported by the observed distribution of core sources in the dataset. Author-, institution-, and country-level productivity patterns were further interpreted using Bradford zones and source growth dynamics. To evaluate RQ5 and RQ6 (authorship patterns and collaborative structures), co-authorship metrics, including single- vs. multi-authored documents, co-authors per article, and collaboration index, were calculated. Co-authorship networks at the author, institution, and country levels were constructed, revealing dense collaboration clusters, country-centered research networks, and extensive multi-country linkages connecting pharmacometrics, machine learning, and systems pharmacology communities. A global distribution of scientific output further highlighted geographic disparities and varying degrees of international collaboration across regions.

For RQ7 (influential documents) and RQ8 (intellectual structure), citation-based analyses, including local/global citation counts, co-citation networks, and bibliographic coupling, were performed to identify seminal contributions that structure the ML-MIDD domain. These analyses provided a comprehensive view of intellectual linkages, enabling interpretation of thematic concentrations and knowledge flows within the field. To address RQ9 and RQ10 (thematic structure and evolution), textual data from author keywords, titles, and abstracts were analyzed to identify conceptual clusters and emerging research fronts. Keyword mapping was performed using VOSviewer (v1.6.20) (Centre for Science and Technology Studies, Leiden University, Leiden, Netherlands) [29], generating (a) network visualizations, (b) overlay visualizations, and (c) heatmaps of keyword occurrences. Word clouds and hierarchical tree maps further supported the identification of key concepts. Thematic maps generated using Biblioshiny enabled classification of themes into motor, basic, niche, and emerging/declining clusters, providing a structured depiction of thematic development and evolution in the ML-MIDD landscape.

To evaluate thematic evolution, the study period (2015–2025) was segmented into three phases based on shifts in topic prominence, frequency, and interconnectedness: (i) early integration (2015–2018), characterized by foundational applications of ML in PK/PD; (ii) method expansion (2019–2021), marked by the emergence of algorithms such as random forest, artificial neural networks, and expanded model-informed workflows; (iii) translational and regulatory adoption (2022–2025), highlighted by themes including personalized medicine, AI-enabled dose optimization, and regulatory-aligned ML workflows. Trend topic analysis and thematic evolution mapping were applied to characterize the transition from traditional pharmacokinetic modeling toward advanced ML-driven precision dosing, drug–drug interaction prediction, virtual clinical trial simulation, and broader applications in quantitative systems pharmacology. Together, these complementary analyses capture both the conceptual structure and the temporal trajectory of the ML-MIDD research landscape.

To formally evaluate whether ML-MIDD publications follow an exponential growth pattern (Price’s Law), annual publication counts were extracted for the period 2016–2025 and analyzed using a logarithmic growth model. Following Price’s formulation, exponential growth is indicated when the natural logarithm of annual publication counts increases linearly over time. Accordingly, the number of publications per year was log-transformed (log_e_ N), and a simple linear regression model of the form log(N) = a + b·Year was fitted using R version 4.5.2 (R Foundation for Statistical Computing, Vienna, Austria). The strength and significance of the linear relationship were assessed through model coefficients, coefficient of determination (R^2^), and 95% confidence intervals. A statistically significant positive slope and strong linear fit indicate conformity with Price’s Law and thus support the presence of exponential growth.

### 2.6. Validation and Reproducibility

All search strategies, database queries, and inclusion criteria were rigorously documented to ensure complete methodological transparency and reproducibility. The raw bibliometric datasets, merged metadata tables, and search outputs were systematically archived to enable independent verification and future reuse. To enhance methodological rigor, the analytical workflow incorporated the PRISMA (Preferred Reporting Items for Systematic Reviews and Meta-Analyses) framework [30], adapted for bibliometric research. While bibliometric analyses emphasize quantitative mapping and network visualization, integrating PRISMA principles provided a systematic validation layer. The framework was applied during dataset screening, inclusion, and refinement to ensure that record selection, deduplication, and synthesis followed transparent, reproducible criteria. The protocol for this bibliometric and systematic review has been prospectively registered in PROSPERO (ID: CRD420251276229). Methodological transparency was ensured through complete documentation of search strategies, screening procedures, data management steps, and analytic workflows, in accordance with PRISMA recommendations. This registration ensures that the review process is publicly accessible and reproducible, providing a reference for verification and future updates.

A structured linkage framework was incorporated to systematically connect bibliometric cluster outputs with the systematic review thematic analysis. Specifically, keyword co-occurrence clusters identified through VOSviewer were mapped to methodological categories by examining (i) dominant algorithmic terms (e.g., neural networks, random forests, PBPK), (ii) application domains (e.g., dose optimization, PK/PD modeling, toxicity prediction), and (iii) study design features reflected in cluster-level metadata. These cluster-derived methodological categories were then used to organize and interpret the systematic review findings, enabling a bidirectional synthesis wherein bibliometric structures informed thematic categorization, and systematic insights contextualized cluster trajectories. A structured PRISMA flow diagram summarized the overall data curation process, including record retrieval, screening, exclusion, and inclusion steps. This diagram complements the bibliometric results by illustrating the transparency and traceability of data management procedures. Through this combined approach, merging quantitative bibliometric mapping with systematic review validation, the study achieved both analytical robustness and methodological transparency. The representative studies were selected using predefined relevance criteria based on (a) adequate sample size or dataset scale, (b) the presence of explicit validation procedures (e.g., cross-validation, external test sets), and (c) clearly reported performance metrics that allowed assessment of methodological rigor. The complete PRISMA checklist used to guide and document this process is provided in Supplementary Data S4.

## 3. Results

### 3.1. Composition and Overlap of the ML-MIDD Publication Landscape

**Figure 1:**
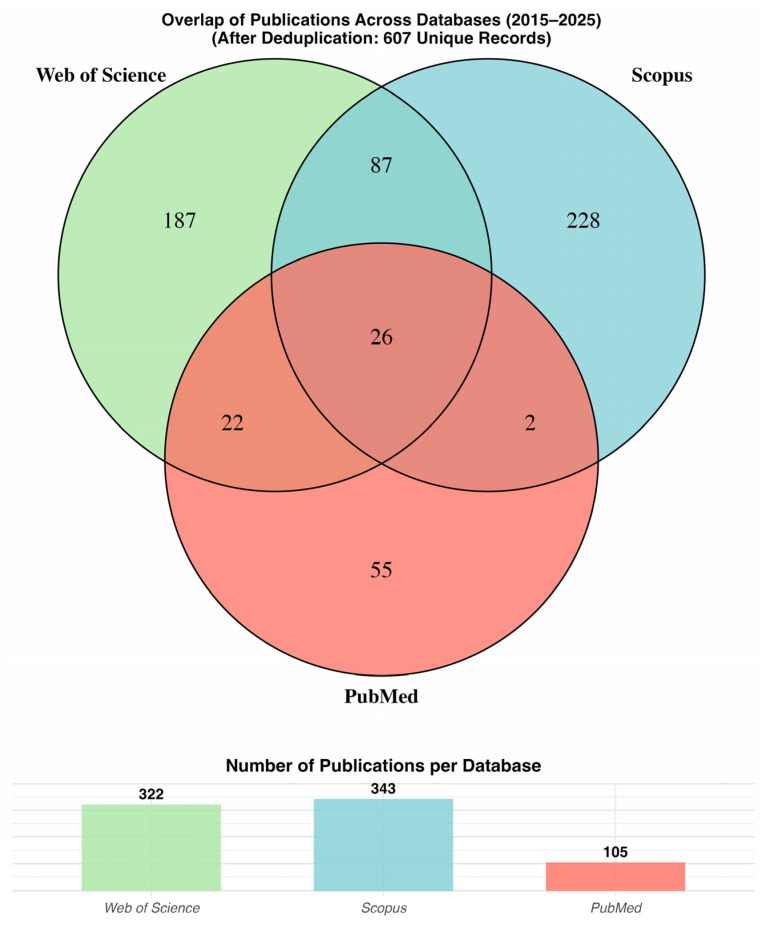
Overlap of Publications Across Databases (2015–2025). Venn diagram illustrating the overlap among bibliographic records retrieved from Web of Science (light green), Scopus (light blue), and PubMed (light red) following data harmonization and deduplication. The figure shows both unique and shared coverage of studies related to the integration of machine learning (ML) with model-informed drug development (MIDD) across major bibliographic databases. A total of 607 unique records were retained after duplicate removal, comprising 187 unique to Web of Science, 228 unique to Scopus, and 55 unique to PubMed. Overlapping records included 87 shared between Web of Science and Scopus, 22 between Web of Science and PubMed, two between Scopus and PubMed, and 26 publications indexed in all three databases. The accompanying bar chart summarizes the total number of records initially retrieved prior to deduplication: 322 from Web of Science, 343 from Scopus, and 105 from PubMed.

A total of 770 records were initially retrieved across three major bibliographic databases, comprising Web of Science (*n* = 322; 41.8%), Scopus (*n* = 343; 44.5%), and PubMed (*n* = 105; 13.6%) (Figure 1). These figures reflect the broader indexing coverage of Scopus and Web of Science for interdisciplinary domains such as machine learning and computational drug development, whereas PubMed, while highly curated for biomedical sciences, retrieved a comparatively smaller proportion of ML-MIDD literature. Following harmonization of metadata fields (e.g., DOIs, titles, authorship, and ISSNs) and systematic deduplication using combined DOI- and title-level matching, the dataset was reduced to 607 unique publications, representing 78.8% of the initial retrieval. This deduplication process eliminated 163 redundant records (21.2%), underscoring the necessity of cross-database integration when conducting bibliometric research in emergent interdisciplinary domains. The Venn diagram clarifies both the overlap and uniqueness of database coverage. The highest degree of bilateral overlap was observed between Web of Science and Scopus (*n* = 87; 14.3% of the final dataset), indicating strong co-coverage among journals indexed by both commercial platforms. Overlap between Web of Science and PubMed accounted for 22 records (3.6%), whereas the shared publications between Scopus and PubMed represented only 2 records (0.3%), highlighting minimal cross-indexing between these sources. Notably, only 26 publications (4.3%) were jointly indexed across all three databases, a nontrivial observation that reflects both disciplinary fragmentation and indexing biases in ML-MIDD research dissemination.

After deduplication, Scopus emerged as the dominant source of unique literature, contributing 228 publications (37.6%), followed by Web of Science with 187 (30.8%), and PubMed with 55 (9.1%). An additional 137 publications (22.6%) were shared between two or more databases. These distribution patterns reveal important differences in database coverage and indexing philosophy. Scopus captures a wide range of computational modeling and pharmaceutical technology research, including journals not indexed in traditional biomedical databases, which likely explains its leading contribution to unique ML-MIDD records. Web of Science shares similarities in structure with Scopus but displays stronger indexing of established pharmacometric and regulatory science journals, contributing high-impact methodological work. PubMed, despite its strengths in biomedical coverage and MeSH-based semantic indexing, contributed only a modest number of unique ML-MIDD records. This finding suggests that a substantial proportion of AI-driven pharmacometrics research is disseminated in interdisciplinary modeling and pharmaceutical informatics journals not exclusively indexed in PubMed.

These findings highlight the methodological necessity of multi-database retrieval in bibliometric studies targeting interdisciplinary research domains. If only Web of Science had been used, 37% of relevant publications would have been lost. This loss would rise to more than 60% if PubMed were used as the sole source. Even Scopus, despite its relatively broad coverage, would omit approximately 25% of relevant publications without cross-database integration. The low three-way overlap (4.3%) is indicative of a highly distributed publication ecosystem, characteristic of emergent interdisciplinary domains where research resides across computational, pharmaceutical, biomedical, and AI-focused journals. This fragmentation is also consistent with the broader trend of ML-related research being disseminated in conference proceedings and non-traditional venues, which may explain partial underrepresentation in biomedical databases such as PubMed. The combined use of multiple databases, coupled with a careful harmonization and deduplication workflow, therefore mitigates systematic retrieval biases while improving the representativeness and reproducibility of bibliometric findings. The resulting dataset of 607 unique ML-MIDD publications provides a robust foundation for subsequent performance indicators, thematic mapping, and evolutionary trend analysis.

### 3.2. Core Journals, Geographical Distribution, and Global Collaboration Patterns

**Figure 2:**
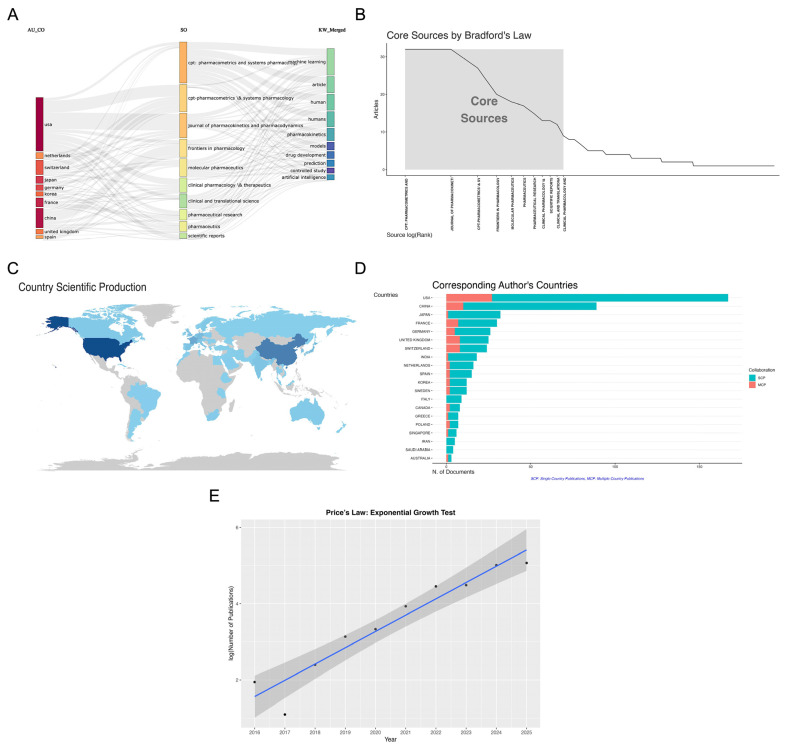
Global publication patterns in machine learning–model-informed drug development (ML-MIDD) research. (**A**) Three-field plot illustrating the relationship between contributing countries (AU_CO), journals (SO), and merged author keywords (KW_Merged). Node colors correspond to different entities within each field: countries (red–orange gradient), journals (yellow–green), and keywords (green–blue), while the thickness of connecting lines represents the relative strength of associations. The connecting lines indicate associations between entities across fields, and line thickness represents the relative strength of association between entities. (**B**) Core sources identified using Bradford’s Law, where the shaded gray region highlights journals classified as the “core zone.” The vertical axis denotes article counts, and the horizontal axis shows the log-rank of each source. (**C**) Global scientific production map showing the geographic distribution of ML-MIDD publications. A blue color gradient legend is used to represent publication volume, where darker blue shades indicate countries with a higher number of publications and lighter blue tones represent lower scientific output. Countries shown in gray indicate no publications retrieved within the analyzed dataset. (**D**) Distribution of corresponding authors by country and their collaboration profiles. Bars are divided into two categories: single-country publications (SCP; green-tosca color) and multiple-country publications (MCP; pink). Bar length reflects the total number of documents attributed to each country. (**E**) Price’s Law exponential growth test based on annual publication counts. The plot displays the logarithm of publication numbers against publication year, with a fitted linear regression line and 95% confidence interval. A linear trend in the log-transformed data indicates exponential growth, supporting the maturation of ML-MIDD as an emerging scientific field.

Figure 2 provides an integrated visualization of the scholarly landscape of ML-MIDD research, encompassing core publication venues, international scientific output, and cross-country collaboration. The three-field plot (Figure 2A) illustrates the linkage between leading contributing countries, top journals, and dominant keywords, revealing the thematic and geographic drivers of the field. The United States appears as the most influential contributor, followed by several European and Asian countries, primarily publishing in high-impact pharmacometric and pharmaceutical science journals and focusing on recurring terms such as machine learning, pharmacokinetics, drug development, and artificial intelligence. This conceptual structure highlights the central role of pharmacometrics-oriented journals in disseminating machine learning-enabled drug development research.

Analysis of journal productivity using Bradford’s Law (Figure 2B) revealed a highly concentrated core of publication sources. The top-ranking journals were CPT: Pharmacometrics and Systems Pharmacology (*n* = 32; 5.1%) and the Journal of Pharmacokinetics and Pharmacodynamics (*n* = 32; 5.1%), followed by CPT-Pharmacometrics & Systems Pharmacology (*n* = 27; 4.3%) and Frontiers in Pharmacology (*n* = 20; 3.2%). Other prominent journals included Molecular Pharmaceutics (*n* = 18; 2.9%), Pharmaceutics (*n* = 17; 2.7%), and Pharmaceutical Research (*n* = 15; 2.4%). Collectively, the top ten journals accounted for approximately 28% of all ML-MIDD publications, demonstrating a high degree of source concentration. While publications span a broad range of pharmaceutical and biomedical journals, the field’s knowledge base remains anchored in pharmacometrics, quantitative pharmacology, and translational modeling. Figure S1 (in Supplementary Data S5) represents a more detailed representation of the most relevant sources. The temporal evolution of journal contributions, illustrated in Figure S2, reveals an inflection point around 2020–2021, with cumulative publications accelerating sharply in the top five journals. CPT: Pharmacometrics & Systems Pharmacology and its sister journal showed the most consistent and rapid growth, particularly after 2021, mirroring the broader adoption of machine learning within regulatory pharmacometrics and clinical trial modeling. Journals such as Frontiers in Pharmacology and Molecular Pharmaceutics also show increasing uptake, reflecting growing interdisciplinary engagement and a widening readership interested in computational approaches to drug development.

In terms of authorship patterns, the most prolific contributors are identified in Figure S3, led by Li Y (16 publications), followed by Woillard J (13), Wang Y (12), Wu Y (12), and Handa K (11). These researchers represent a blend of pharmacometricians, clinicians, and computational scientists, underscoring the interdisciplinary nature of ML-MIDD. The temporal publishing trajectories presented in Figure S4 reveal that many of these authors have only begun to publish extensively in the last 3–5 years, indicating a relatively recent intensification of expertise and leadership in the field. The most active authors demonstrate sustained output and increasing citation impact over time, suggesting emerging thought leadership and research consolidation within a growing scientific niche.

Citation analysis of influential publications (Figure S5) highlights both foundational and methodological papers driving the field. The most globally cited article is by Badillo et al. (2020, Clinical Pharmacology & Therapeutics), with 303 citations [31], followed by Fan et al. (2019, Medical Physics, 278 citations) [32] and Greffier et al. (2020, European Radiology, 254 citations) [33]. These highly cited works often represent methodological advancements, reviews, or exemplary ML applications with demonstrated impact on clinical or translational decision-making. Notably, several high-impact articles were published within the last five years, further evidence of the rapid influence and knowledge dissemination in the ML-MIDD domain. Institutional analysis (Figure S6) shows that academic and industry collaboration is equally important. The most productive affiliation is the National University of Singapore (35 publications), followed by the University of Florida (24), Genentech, Inc. (23), LabRiffe (23), and the University of Limoges (23). The inclusion of major pharmaceutical companies and regulatory-oriented institutions among the top contributors highlights the translational and implementation-driven focus of ML-MIDD research. Other leading institutions include Fudan University, Army Medical University, and Roche Innovation Center Basel, reflecting both geographic diversity and growing participation from Asia and Europe. These institutional patterns indicate increasing alignment between academic research, clinical pharmacology groups, and industry stakeholders in advancing model-based ML applications.

The geographical distribution of contributing authors (Figure 2C) indicates that the United States leads ML-MIDD research (*n* = 665; 20.8%), followed by China (*n* = 394; 12.3%), France (*n* = 180; 5.6%), Japan (*n* = 99; 3.1%), and Germany (*n* = 92; 2.9%). Notable additional contributors include Switzerland (n = 89; 2.8%), South Korea (n = 72; 2.3%), and the United Kingdom (*n* = 72; 2.3%). Several emerging contributors from Europe and the Asia-Pacific region, including Spain, Italy, India, and Singapore, each contributed between 1.5–2.0% of total publications. Countries such as South Africa (*n* = 15; 0.5%) and Iran (*n* = 13; 0.4%) also demonstrated active participation, reflecting growing global adoption of computational pharmacology and AI-driven drug development methodologies. Longitudinal trends (Figure S7) show a marked rise in research activity from 2019 onward, with the United States exhibiting the strongest increase and surpassing 600 cumulative articles by 2025. China displayed the second-largest expansion, reaching nearly 400 publications by 2025, while France, Germany, and Japan showed more moderate but steady growth over the same period.

Corresponding author analysis (Figure 2D) further confirms the dominance of the United States (*n* = 167; 27.5%) and China (*n* = 89; 14.7%). Other high-output countries included Japan (*n* = 32; 5.3%), France (*n* = 30; 4.9%), Germany (*n* = 26; 4.3%), and the United Kingdom (*n* = 25; 4.1%). Switzerland, India, the Netherlands, and Spain contributed between 2.4–4.0% each. Moderate output was also observed from South Korea and Sweden (each *n* = 12; 2.0%), Italy (*n* = 9; 1.5%), and Canada (*n* = 8; 1.3%). Only a small fraction of corresponding authorship was contributed by countries such as Iran (*n* = 5; 0.8%), Saudi Arabia (*n* = 4; 0.7%), and Australia (*n* = 3; 0.5%). Multiple-country publications (MCP) represented a substantial proportion of output from leading countries, reflecting strong international collaboration. The United States, China, and several European nations demonstrate balanced contributions from both single-country publications (SCP) and cross-border research, underscoring the globalized and multidisciplinary nature of ML-driven drug development. Citation impact analysis (Figure S8) further reinforces the central influence of the United States, which accumulated the highest number of citations (*n* = 2871), followed by China (*n* = 884), France (*n* = 620), and Switzerland (*n* = 505). Germany (*n* = 507), Japan (*n* = 501), and the United Kingdom (*n* = 474) also demonstrated strong citation performance despite lower publication volume, indicating high-impact contributions. Countries such as Sweden (*n* = 185) and Israel (*n* = 185) ranked within the top 10 by citation count, suggesting a selective but influential research output.

Figure 2E presents the results of Price’s Law exponential growth test based on annual publication counts from 2016 to 2025. Over this ten-year period, the number of ML-MIDD publications increased from 7 articles in 2016 to 159 in 2025, representing more than a 22-fold increase and highlighting a marked acceleration in scientific output. When the annual publication counts were log-transformed (log_e_N), the resulting values aligned closely with a linear trend across the examined years, confirming that growth in ML-MIDD research adheres to an exponential trajectory, in accordance with Price’s Law. The fitted linear model demonstrated a strong goodness of fit, with the regression line closely tracing the log-transformed data points, and the accompanying 95% confidence interval remained narrow across most years, indicating stability and robustness of the estimated growth rate. This pattern reflects a sustained and systematic expansion of the field rather than sporadic or irregular increases in publication activity. The upward slope of the regression line quantifies the intrinsic growth rate of ML-MIDD literature, suggesting that the field is transitioning from an early developmental phase into a more rapidly maturing scientific domain. The evidence of exponential growth provided by Price’s Law analysis offers a quantitative basis for characterizing ML-MIDD as an emerging research area undergoing accelerated expansion. This strengthens the rationale for conducting both a bibliometric mapping and a systematic review, as such exponential growth typically accompanies diversification of research themes, increased interdisciplinarity, and the establishment of stable collaborative networks.

### 3.3. Thematic Structure, Keyword Dynamics, and Evolution of Research Trends

**Figure 3:**
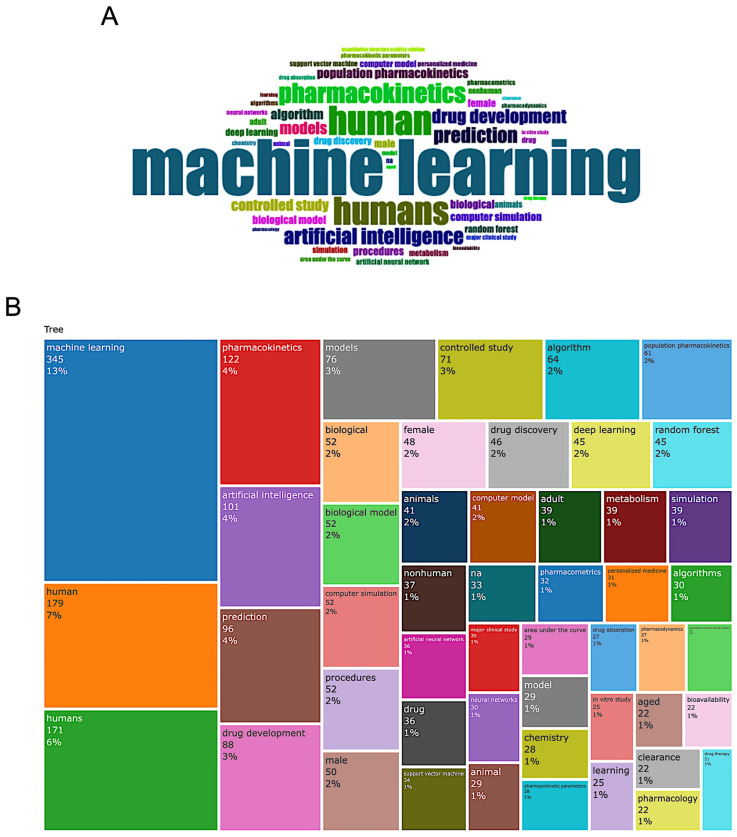
Visual representation of high-frequency keywords in the machine learning–model-informed drug development (ML-MIDD) literature. (**A**) Keyword word cloud illustrating the relative prominence of author keywords; larger font size corresponds to higher keyword frequency, and colors are automatically assigned by Biblioshiny solely for visual separation without representing any analytic grouping. (**B**) Keyword Treemap displaying the most frequently occurring keywords, where each rectangle’s size is proportional to keyword frequency, and colors are assigned by the software to distinguish blocks and do not represent thematic categories. Numeric labels indicate the frequency and percentage contribution of each keyword within the overall dataset.

The evolution of research topics in ML-MIDD is reflected through both the structural mapping of frequently used keywords and the temporal dynamics of their growth. Figure 3 illustrates the conceptual landscape of ML-MIDD research by visualizing the most frequently occurring keywords using a word cloud (Figure 3A) and a hierarchical keyword TreeMap (Figure 3B). Across the dataset of 607 unique articles, “machine learning” dominates as the central research theme (*n* = 345 occurrences; 13% of total keyword mentions), underscoring its foundational role in this domain. Because the keyword visualizations were generated directly in Biblioshiny, which automatically extracts and displays author keywords, Keywords Plus, and indexed terms without allowing manual editing, some semantically similar terms (e.g., “human” and “humans”) appear as separate entries. Other high-frequency terms include “human” (*n* = 179; 7%), “humans” (*n* = 171; 6%), and “pharmacokinetics” (*n* = 122; 4%), reflecting the intersection of computational approaches and clinical pharmacology. The prominence of “drug development” (*n* = 88; 3%), “models” (*n* = 76; 3%), and “controlled study” (*n* = 71; 3%) highlights the importance of predictive modeling, experimental design, and applied translational research in this field. Keywords such as “artificial intelligence” (*n* = 101; 4%), “prediction” (*n* = 96; 4%), “deep learning” (*n* = 45; 2%), “random forest” (*n* = 45; 2%), and “neural networks” (*n* = 30; 1%) reveal the diverse methodological spectrum and the ongoing shift from traditional statistical modeling toward more advanced AI-based analytical frameworks. The TreeMap offers additional thematic granularity, revealing clusters around pharmacometric concepts and model-based methodologies, such as “population pharmacokinetics” (*n* = 61; 2%), “biological model” (*n* = 52; 2%), “computer simulation” (*n* = 52; 2%), and “drug discovery” (*n* = 46; 2%). The frequent appearance of demographic descriptors, such as “male” (*n* = 50; 2%), “female” (*n* = 48; 2%), and “adult” (*n* = 39; 2%), suggests the continued integration of mechanistic and ML models with patient-level clinical data, supporting precision medicine initiatives. Additionally, terms like “metabolism” (*n* = 39; 2%), “clearance” (*n* = 22; 1%), “drug absorption” (*n* = 27; 1%), and “bioavailability” (*n* = 22; 1%) indicate strong alignment with PK/PD modeling and model-informed regulatory strategies, despite the presence of non-normalized keyword variants inherent to automated bibliometric tools.

**Figure 4:**
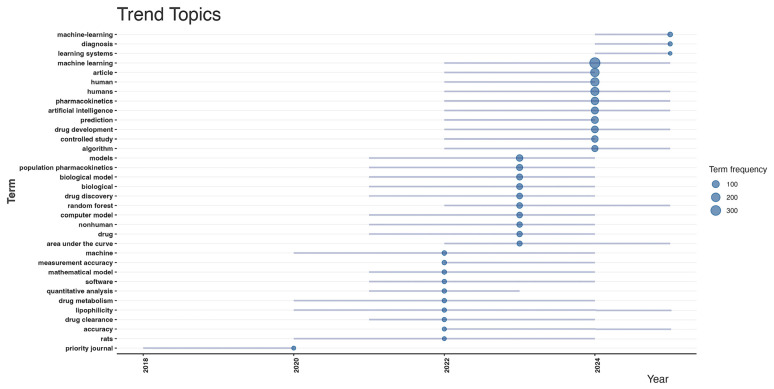
Temporal evolution of research topics in machine learning–model-informed drug development (ML-MIDD). Trend topic analysis generated using Biblioshiny, illustrating when high-frequency keywords emerged and how long they remained prominent in the literature. Each horizontal line represents the active period of a keyword, while bubble size reflects the relative frequency of the term (larger bubbles = higher occurrence). All terms are shown in blue, as automatically assigned by Biblioshiny, and bubbles represent cumulative frequency across the dataset. The x-axis indicates the average publication year during which each keyword was most prevalent.

Emerging research trends are further contextualized through temporal topic analysis, shown in Figure 4. Early-stage studies (2016–2019) were dominated by general methodological terms such as “machine learning,” “diagnosis,” “learning systems,” and “algorithms.” These early topics reflect foundational work establishing ML approaches within pharmacology and clinical research. From 2020 onward, there has been substantial thematic diversification, with increased activity in topics explicitly related to pharmacometrics, including “population pharmacokinetics,” “biological model,” “random forest,” “drug discovery,” “drug clearance,” “drug metabolism,” and “mathematical model.” More recent trends (2023–2025) indicate a pivot toward applied therapeutics and model integration, with increasing attention to “measurement accuracy,” “quantitative analysis,” “area under the curve,” “lipophilicity,” and “software.” These shifts demonstrate the evolution of ML-MIDD from a conceptual framework to a more implementation-driven, mechanistically integrative research paradigm. The keyword-based analyses emphasize that ML-MIDD has transitioned from exploratory adoption of ML tools to increasingly sophisticated applications in PK/PD modeling, therapeutic optimization, and translational decision-making. The presence of both methodological and biomedical keywords confirms the interdisciplinary nature of the field and underscores its progression toward regulatory and clinical relevance.

Figure S9 further reinforces these observations by presenting cumulative keyword occurrences from 2016 to 2025. The steep and consistent rise in keywords such as machine learning, artificial intelligence, pharmacokinetics, drug development, prediction, and models reflects a broad expansion in both methodological and domain-specific research applications. Notably, the curve for “machine learning” increases more rapidly than any other term, underscoring its central role within the MIDD landscape. The parallel rise of “artificial intelligence” and “prediction” indicates that ML approaches are increasingly being adopted not only for descriptive analysis but also for predictive modeling and decision support in drug development pipelines.

**Figure 5:**
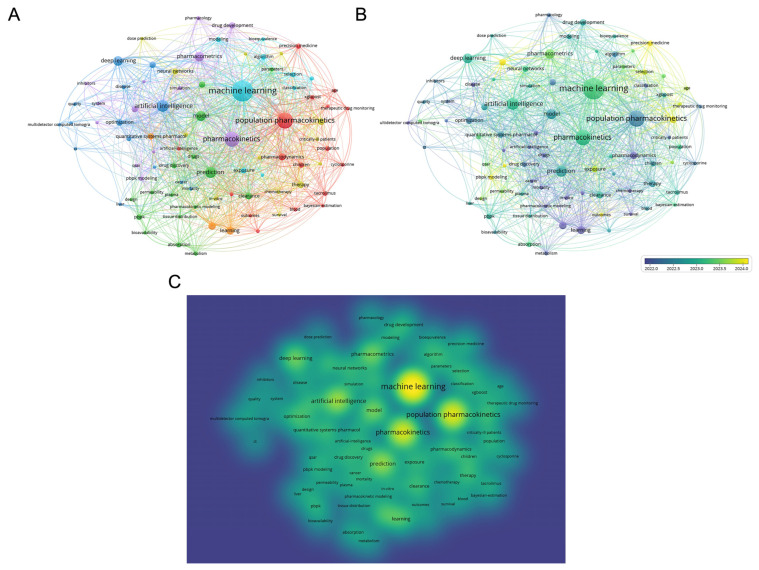
Keyword co-occurrence and temporal distribution of research topics in machine learning–model-informed drug development (ML-MIDD). (**A**) Network visualization map of author keyword co-occurrences generated using VOSviewer. Nodes represent distinct keywords, where node size reflects keyword frequency and node color indicates cluster membership, with each cluster representing a group of closely related research themes. Thicker connecting lines represent stronger co-occurrence relationships. (**B**) Overlay visualization showing the temporal evolution of keyword usage based on the average publication year. The color gradient ranges from dark blue (earliest years) to yellow (most recent years), highlighting emerging research areas and recent thematic shifts. Node size again corresponds to keyword frequency. (**C**) Density heatmap visualization illustrating the concentration and clustering of high-frequency keywords. Warmer colors (yellow–green) indicate regions with higher keyword density, while cooler blue areas represent zones of lower occurrence.

**Figure 6:**
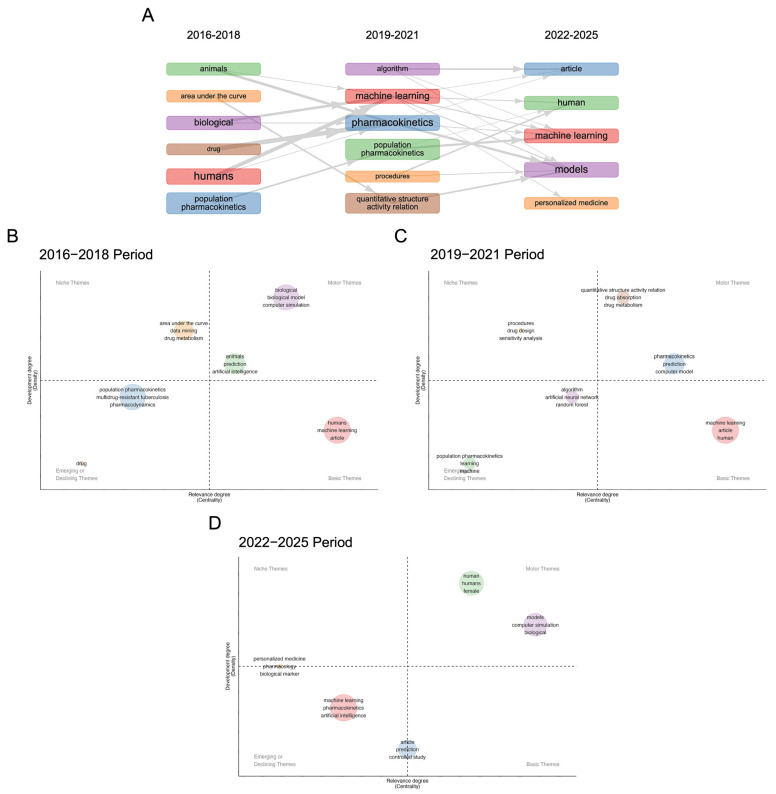
Thematic structure and evolution of machine learning-model-informed drug development (ML-MIDD) research. (**A**) Thematic evolution map showing conceptual shifts across three time periods based on the top 250 most frequent keywords. Arrows represent the continuity of themes between consecutive periods, and arrow thickness reflects the number of shared keywords carried forward, with thicker arrows indicating stronger thematic continuity. (**B**) Thematic map for the 2016–2018 period. (**C**) Thematic map for the 2019–2021 period. (**D**) Thematic map for the 2022–2025 period. Each map is divided into four quadrants: Upper-right (Motor Themes): highly developed and central themes (high density, high centrality). Upper-left (Niche Themes): specialized but isolated themes (high density, low centrality). Lower-right (Basic Themes): fundamental, widely connected themes (low density, high centrality). Lower-left (Emerging or Declining Themes): weakly developed or transitional themes (low density, low centrality). Node size reflects the frequency of each keyword within its respective period, and node color represents the cluster to which each keyword belongs.

To gain deeper insights into the intellectual structure and temporal progression of ML-MIDD research, keyword co-occurrence networks (Figure 5) and thematic evolution maps (Figure 6) were constructed. These analyses allow visualization of core research foci, conceptual linkages, and shifts in thematic prominence over time. The VOSviewer keyword network (Figure 5A) reveals a highly interconnected research landscape anchored around several central concepts. Machine learning forms the dominant and most influential node, closely linked to terms such as population pharmacokinetics, pharmacokinetics, artificial intelligence, prediction, deep learning, and drug development. These clusters reflect the convergence of pharmacometric modeling and algorithmic approaches toward predictive, data-driven decision-making in drug development and therapeutic individualization. Peripheral clusters highlight specialized subdomains such as physiologically based pharmacokinetic (PBPK) modeling, precision medicine, drug exposure, and dose optimization, indicating emerging specialization beyond foundational ML and PK methods.

The overlay visualization (Figure 5B) illustrates a clear temporal progression in the field. To facilitate interpretation of this progression, the timeline was segmented into three descriptive periods (2016–2018, 2019–2021, 2022–2025). This segmentation was not derived from formal statistical breakpoint or change-point detection, but was instead informed by observed shifts in publication density, keyword prominence, and thematic structure across the dataset. External technological and regulatory milestones were considered only as contextual reference points, rather than as empirically demonstrated causal drivers of the observed trends. Specifically, the 2016–2018 period corresponds to an early phase of ML-MIDD research characterized by relatively low publication volume and predominantly exploratory applications, temporally overlapping with the initial dissemination of widely adopted deep learning frameworks, including TensorFlow (v1.0, released in 2017) and PyTorch (early public releases v0.1–v1.0, 2017–2018). However, we do not claim a direct causal relationship between these software releases and changes in ML-MIDD research output. The 2019–2021 interval aligns with a visible increase in publication volume and thematic diversification observed in the bibliometric data, which temporally coincides with broader access to high-performance computing resources and the COVID-19 pandemic. These external events are discussed as plausible contextual factors rather than as quantitatively validated inflection drivers, acknowledging that the observed growth may reflect multiple converging influences, including increased data availability, methodological maturation, and heightened interest in in silico drug development [34,35]. Finally, the 2022–2025 period reflects a sustained increase in publication output and consolidation of ML-centric themes, alongside growing regulatory discussion of model-informed drug development and ML-enabled tools. This period is defined by data-observed thematic stabilization rather than by prespecified regulatory milestones, although contemporaneous regulatory initiatives provide relevant contextual background [36,37]. The heatmap visualization (Figure 5C) supports this interpretation, with dense keyword co-occurrence hotspots centered on machine learning, population pharmacokinetics, and artificial intelligence, marking these as dominant conceptual drivers during later years.

The thematic evolution analysis (Figure 6A) further contextualizes these patterns by illustrating how research themes emerged, matured, or declined across the three analytically defined periods. During 2016–2018, basic themes were largely methodological or domain-rooted (e.g., population pharmacokinetics, drug metabolism, biological models) (Figure 6B), while machine learning-related concepts remained peripheral. This pattern reflects an exploratory stage of ML-MIDD research rather than a clearly defined structural shift. In the 2019–2021 period, machine learning and algorithms moved toward the motor theme quadrant (Figure 6C), indicating increased centrality and internal development. This transition is empirically observed in the thematic maps but is not attributed to a single triggering event. Concurrently, procedures, drug design, and sensitivity analysis gained prominence, suggesting diversification toward workflow automation and optimization.

In the most recent period (2022–2025), machine learning and humans occupy the most central and developed thematic space (Figure 6D), confirming consolidation of ML approaches in translational and patient-focused contexts. Personalized medicine, pharmaco-omics, and biological markers emerge as developing or niche themes, indicating increasing emphasis on integrating multi-omics, clinical, and real-world data. Conversely, earlier themes such as animals and in vitro study decline in prominence, reflecting a shift toward clinically oriented, model-informed, and computational strategies. Thus, the three-period segmentation should be interpreted as an analytical framework to summarize observed temporal patterns, rather than as evidence of discrete, causally defined phases. Formal statistical identification of natural breakpoints represents an important direction for future bibliometric and topic-evolution analyses in ML-MIDD.

### 3.4. Integrative Systematic Review Using PRISMA to Refine Bibliometric Findings

**Figure 7:**
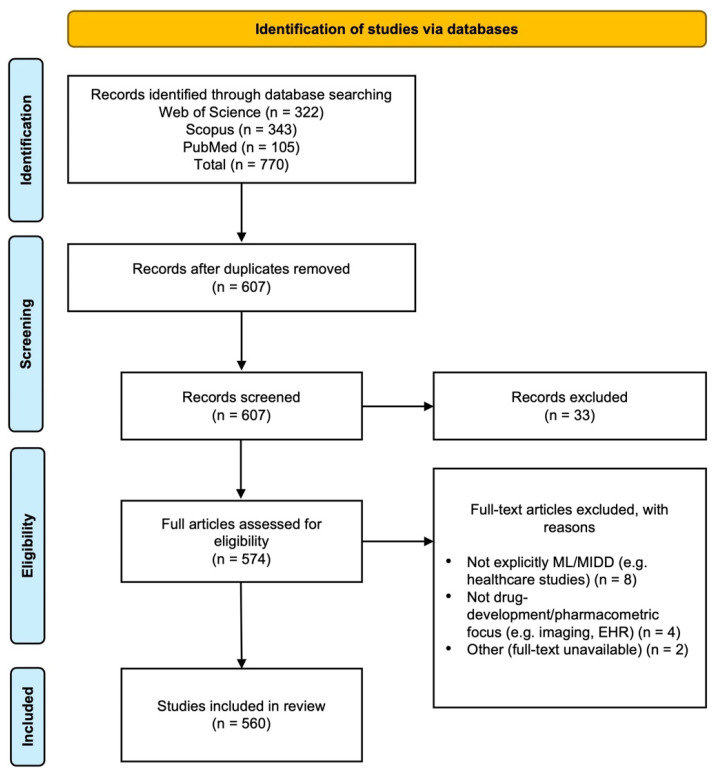
The PRISMA flowchart illustrates the step-by-step study selection process, from initial record identification through screening and eligibility assessment, showing the number of studies excluded at each stage and ultimately included. This process complements the bibliometric clusters by mapping the methodological breadth and maturity of machine learning-model-informed drug development (ML-MIDD) research.

A systematic review was employed to further refine and contextualize the findings from the bibliometric analysis by systematically identifying, evaluating, and mapping relevant literature at the ML-MIDD interface. This step was designed to explicitly link bibliometric clusters, such as reinforcement learning applications, ML-PBPK modeling, hybrid mechanistic/ML approaches, and generative AI frameworks, to methodological approaches identified in the systematic review, thereby bridging the two parallel analyses. A total of 770 records were retrieved from three major bibliographic databases: Web of Science (*n* = 322; 41.8%), Scopus (*n* = 343; 44.5%), and PubMed (*n* = 105; 13.6%). After removal of duplicates derived from the bibliometric workflow, 607 unique records remained and proceeded to the title and abstract screening stage. Of these, 33 records were excluded (*n* = 33; 5.4%), primarily due to lack of relevance to ML-MIDD or their focus on domains outside drug development. A total of 574 full-text articles were then assessed for eligibility. During this stage, additional records were excluded for the following reasons: not explicitly ML-MIDD (*n* = 8; 1.4%), such as generic healthcare AI studies without modeling relevance; not drug-development or pharmacometric focused (*n* = 4; 0.7%), including studies centered on imaging or electronic health record analytics without modeling components; and other reasons (*n* = 2; 0.3%), including lack of full-text availability or non-English language. After applying these predefined inclusion and exclusion criteria, 560 studies (*n* = 560; 97.6% of screened full texts) were included in the final systematic review (Figure 7).

The final body of literature reflects the breadth and maturity of ML-MIDD research, encompassing a wide variety of methodologies, modeling frameworks, therapeutic areas, and development stages. To better integrate with the bibliometric analysis, we mapped key bibliometric clusters to representative methodological approaches identified in the systematic review. For instance, studies within the “reinforcement learning” cluster correspond to precision dosing applications, while “ML-PBPK” and “QSP hybrid models” clusters correspond to pharmacokinetic/pharmacodynamic parameter prediction and systems pharmacology modeling, respectively. To highlight the most relevant and impactful work at this emerging interface, Table 1 presents a set of representative studies illustrating ML-enabled advancements across MIDD workflows. These include the use of reinforcement learning for precision dosing, integration of radiomics and ML for model-informed personalization, ML-enhanced PBPK and QSP applications, hybrid mechanistic/data-driven modeling approaches, and the application of generative AI and large language models in QSP and pharmacometric workflows. Where available, we also report dataset characteristics, sample sizes, validation approaches, and key performance metrics (e.g., R^2^, reduction in dosing error, computational efficiency) to provide context for methodological rigor and reproducibility. While Table 1 focuses on illustrative exemplars, the complete list of curated studies is available in Supplementary Data S6. The selection process confirms the rapid growth of research bridging machine learning with model-informed drug development. The included studies demonstrate increasing methodological sophistication, expanded application in decision support, and a clear shift toward integrative, automated, and data-centric paradigms that complement traditional pharmacometric modeling frameworks.

## 4. Discussion

Recent years have witnessed a notable shift in quantitative pharmacology as ML tools become increasingly integrated with traditional mechanistic models. Our analysis of the 2015–2025 literature highlights three mutually reinforcing trends: (1) rapid maturation of ML methods tailored to pharmacometrics (e.g., covariate selection algorithms, ML-based surrogate models, and hybrid mechanistic/ML architectures); (2) broader uptake of ML across the entire MIDD pipeline (from drug discovery through formulation and clinical trial design); and (3) growing focus on validation and regulatory requirements needed for ML models to achieve decision-grade reliability. These themes echo recent reviews noting both the promise and challenges of ML in quantitative pharmacology and regulatory science [50]. In particular, hybrid or physics-informed ML approaches, which preserve mechanistic interpretability while harnessing ML for high-dimensional pattern learning, have emerged as a pragmatic pathway to stronger predictions and regulatory acceptance.

Importantly, because this study employed explicit inclusion and exclusion criteria, such as limiting records to original research and reviews published between 2015 and 2025, restricting sources to Web of Science, Scopus, and PubMed, and requiring explicit relevance to machine learning or model-informed drug development, these methodological boundaries shaped both the scope and the trends observed. The emphasis on peer-reviewed, English-language documents and the requirement for clear ML–MIDD linkage necessarily excluded conference abstracts, non-indexed regional publications, and grey literature. As a result, some geographic research contributions (especially from regions with lower indexation coverage) may be underrepresented, and the thematic landscape may be biased toward topics more frequently published in high-visibility journals. These implications are further elaborated below.

### 4.1. Methodological Advances and Hybrid Modeling

The methodological frontier is moving from isolated ML case studies toward hybrid modeling frameworks that combine mechanistic knowledge with data-driven components. For example, Jia et al. (2025) introduced a hybrid ML-PBPK model in which a neural network acts as a fast surrogate within a PBPK workflow [51]. In their approach, the ML component learns from large PK datasets while the PBPK structure retains physiological interpretability; the hybrid model achieved two-fold accuracy for the majority of 106 test drugs. Similarly, Androulakis et al. (2025) note that modern ML enables the creation of digital twin-style frameworks, in which surrogate models and hybrid networks quickly simulate PK/PD systems [52]. In these models, ML is used to ‘collapse’ costly simulations into learned emulators, allowing virtual trials to run orders of magnitude faster. Moreover, generative models (e.g., VAEs) are being adapted to NLME problems: recent work demonstrates that a VAE can jointly estimate population parameters and select covariates “all at once” in a single run [48]. In essence, the VAE learns a latent representation of the NLME model and optimizes covariate inclusion simultaneously, obviating the need for iterative covariate-search loops.

This trend toward hybridization is motivated by a desire to maintain mechanistic interpretability while leveraging ML’s flexibility. As Terranova et al. (2024) emphasize, embedding a priori domain knowledge (physics, pharmacology, etc.) into ML architectures can greatly improve model generalizability and reliability [53]. Approaches like geometric deep learning or pharmacology-informed neural networks explicitly encode invariances or known causal structures so that the ML model respects underlying biological constraints. Not only do these hybrid and scientific ML methods tend to improve predictive performance, but they also help satisfy regulatory expectations for interpretability. To ensure the mechanistic components remain valid when embedded within ML architectures, hybrid models are increasingly evaluated using a combination of internal simulations, external datasets, and sensitivity or uncertainty analyses focused specifically on the mechanistic parameters. Model verification steps may include comparing predictions from the mechanistic core against known physiological benchmarks or clinical measurements, while ML surrogates are assessed for consistency across multiple folds of cross-validation [54,55]. In sum, the literature suggests that the most impactful advances will likely come from coupling mechanistic models with ML surrogates or parsers, rather than treating ML as a purely black-box replacement. Recent reviews have similarly concluded that “physics-informed” and hybrid ML models are among the most promising ways to scale complex PK/PD models to large biomedical datasets while preserving insight and credibility [52,53].

### 4.2. Applications Across the Development Pipeline

ML methods are now being applied at every stage of drug development. In early discovery, ML accelerates compound screening and target prioritization. For example, D-MPNNs and other QSAR-style models are increasingly used to predict ADME or toxicity endpoints, triaging candidates before costly lab tests [42]. In formulation development, neural networks and other ML models are used to optimize excipient compositions and predict in IVIVC [49]. A study reviews numerous studies where ANNs predict dissolution profiles or tablet release characteristics, enabling rapid in silico formulation screening and virtual bioavailability experiments [47]. In PBBM, neural-network surrogates are embedded to accelerate the simulation of complex release and absorption processes, thereby facilitating high-throughput virtual bioequivalence studies. During clinical development, ML augments traditional trial simulations and analysis. For instance, tree-based ML or neural network models have been shown to predict individual PK profiles comparably to PopPK models. That study reports that popular ML algorithms (random forests, gradient boosting, neural nets) achieve drug-concentration predictions on par with NLME models, especially when combined in ensembles [50]. In adaptive trial design, reinforcement-learning and decision-theoretic methods are emerging for individualized dosing and dose-finding strategies. Though still nascent in formal pharmacometrics, pilot studies apply RL to recommend dose adjustments or patient allocation in simulations. Overall, the breadth of ML applications mirrors what others have documented. A study explicitly outlines AI/ML use cases across pharmacometrics and systems pharmacology, clinical trial modeling, and even NLP-driven literature mining [53]. Likewise, Androulakis et al. (2025) highlight how ML can automate literature review and translate data into model components, effectively creating digital-twin templates for patient populations [52]. These surveys underscore that ML is reducing cycle times (e.g., by replacing animal PK/PD studies with virtual experiments) and enabling in silico MBMA (model-based meta-analysis) and QSP analyses that were previously too labor-intensive. However, because our inclusion criteria prioritized publications with explicit ML–MIDD methodological integration, studies applying ML to broader pharmaceutical domains without a clear pharmacometric or modeling component were excluded. This decision ensured conceptual coherence but may have narrowed the representation of emerging ML applications that fall adjacent to, but not directly within, MIDD. Similarly, the exclusion of non-indexed preprints and conference proceedings may delay the visibility of very recent innovations, particularly in fast-moving ML subfields.

### 4.3. Regulatory Context, Validation, and Transparency

Despite rapid methodological progress, a recurring theme is the rigor of validation and documentation needed for regulatory acceptance. Regulatory bodies and industry experts repeatedly caution that ML-enhanced MIDD models must meet the same evidentiary standards as traditional mechanistic models. Importantly, while MIDD frameworks are increasingly recognized in regulatory science, formal regulatory guidance on ML-MIDD integration is still emerging, and few ML-augmented approaches have been explicitly accepted in submissions. For example, a study reviews VBE methods and notes that “regulatory acceptance of virtual bioequivalence relies heavily on the validation of computational models and the availability of accurate, comprehensive data inputs” [10]. In practice, this means ML components in a modeling workflow should have pre-specified validation datasets, robust sensitivity/uncertainty quantification, and demonstrated external generalizability. Case studies exist where ML-enabled components have been applied within regulatory submissions as supportive elements of established MIDD workflows, such as AI-assisted or data-driven PBPK modeling frameworks used to support dose selection in oncology or pharmacogenomic-informed analyses cited in FDA review documents. Importantly, these examples reflect indirect or adjunctive use of ML techniques rather than explicit regulatory acceptance of ML-MIDD as a formal methodology, demonstrating that ML can complement traditional regulatory frameworks when validation and transparency are sufficient [20,56,57]. Recent regulatory reviews highlight the presence of ML-augmented or algorithm-assisted modeling components in drug applications. For example, the FDA’s review of Sotorasib (AMG 510, a KRAS G12C inhibitor) used PBPK modeling to support the 960 mg dose; while ML-derived tools may have contributed to model development or parameter screening, the regulatory decision was grounded in conventional PBPK principles, consistent with the agency’s “Project Optimus” focus on dose optimization [58,59]. Likewise, the FDA clinical pharmacology review of Abemaciclib (Verzenio) describes model-based assessments of drug–drug interaction risk that were aided by algorithmic covariate screening methods (a form of ML-based feature selection) to identify key predictors, without constituting a formal ML-MIDD regulatory framework [60,61]. In Europe, the EMA’s Lorviqua (Lorlatinib) assessment similarly incorporated advanced model-informed exposure predictions supported by data-driven (ML) components. Even the FDA’s MIDD Paired-Meeting case studies cite examples of ML-assisted PBPK modeling and ML-based covariate screening as enabling tools for pediatric dose optimization and oncology exposure–response analyses. Collectively, these cases illustrate cautious and context-dependent use of ML within established MIDD paradigms, rather than broad regulatory endorsement of ML-MIDD as an independent decision-making methodology [58,62].

The IQ Consortium white paper similarly recommends that performance metrics be tailored to the context of use, and that models demonstrate good generalizability outside the training domain. In short, “beware of potential overfitting,” it warns, and ensure the AI/ML model is tested against independent data and meets regulatory-grade reliability [53]. Another consistent message is that transparency and explainability are essential. Complex ML architectures are often seen as “black boxes,” so regulators will want clear documentation of how inputs are processed and why predictions are made. Regulatory guidance increasingly emphasizes traceability of model decisions, comprehensive reporting of training data, assumptions, and limitations, and inclusion of model verification and validation (V&V) activities aligned with Good Modeling Practice (GMP) principles [63].

Explainable AI (XAI) techniques, such as feature attribution methods like SHapley Additive exPlanations (SHAP) and Local Interpretable Model-agnostic Explanations (LIME) or counterfactual explanations, are increasingly applied to pharmacometric models so that ML recommendations can be interpreted in pharmacological terms. A study emphasizes that achieving trust in medical AI requires a holistic approach to transparency “from data collection and model development to clinical deployment,” and that XAI tools (feature importance, counterfactuals, concept-based methods) should be used to make model reasoning visible [64]. These tools help investigators verify that key PK or patient covariates influence predictions sensibly, which aids both scientific understanding and regulatory review. Our exclusion of studies lacking methodological transparency or missing sufficient model detail, criteria applied to ensure scientific rigor, may have limited the representation of ML studies that are clinically relevant but less explicit in documenting model architectures and workflows. Although this improves the reliability of the included evidence, it inherently filters out early-stage, exploratory ML applications that may influence future regulatory discussions. Thus, while ML-MIDD is a promising regulatory science tool, widespread regulatory acceptance requires further case studies, standardized validation frameworks, and clear documentation to ensure alignment with current regulatory expectations.

## 5. Limitations and Future Works

This study provides an integrated quantitative and qualitative assessment of emerging trends at the intersection of ML and MIDD, yet several limitations should be acknowledged. These limitations primarily concern the scope of literature integration, methodological constraints inherent to bibliometric and systematic review approaches, and evolving definitions of ML and MIDD that influence inclusion criteria. These factors may impact the comprehensiveness of the insights and generalizability of the findings. Despite these challenges, the current review offers a foundational perspective that can guide future investigations and methodological innovations in this dynamic area of research.

### 5.1. Limitations

The first limitation of this study stems from the database selection and search strategy. While three major bibliographic sources were used (Web of Science, Scopus, and PubMed), relevant non-indexed or grey literature (e.g., preprints, regulatory submissions, conference proceedings) may have been excluded, potentially underrepresenting emerging or industry-specific contributions. Additionally, the analysis was restricted to English-language publications, which may have omitted valuable studies published in other languages. The bibliometric methodology also relies heavily on the accuracy of indexing and metadata, which can lead to misclassification of fields or underrepresentation of interdisciplinary works.

Secondly, while the systematic review component refined the bibliometric output to identify conceptually significant studies, the absence of a quantitative quality assessment introduces subjectivity in the inclusion process. The broad definitions of ML and MIDD used in the literature also present challenges; for example, works implementing statistical learning or simple regression frameworks may be inconsistently classified as ML across studies. Moreover, the rapidly evolving nature of the field means that studies published after the search cutoff or those in early dissemination stages (e.g., preprints) may not be captured, limiting the temporal comprehensiveness of the review. Finally, the heterogeneity of methodologies and reporting standards across the included studies complicates direct comparison and synthesis. The variability in model types, data sources, therapeutic contexts, and validation strategies limits the capacity to perform meta-analysis or quantitative synthesis beyond the descriptive mapping provided.

### 5.2. Future Works

Future research should build upon these findings by focusing on the scientific and translational advancement of ML-MIDD, including expanding the scope and resolution of analytical frameworks used to assess ML influence across MIDD. Incorporating additional data sources (e.g., ClinicalTrials.gov, FDA submissions, patent databases, preprints, or industry reports) could provide a more complete picture of translational and regulatory adoption. Systematic inclusion of grey literature and non-English studies may also help capture global diversity in ML-MIDD applications.

Further, advancing this work will require deeper quantitative analyses, including performance benchmarking of ML versus traditional approaches across specific MIDD tasks (e.g., covariate modeling, virtual bioequivalence, adaptive dose selection). Longitudinal studies tracking citation patterns, method adoption, and regulatory milestones over time could provide insight into how ML-enabled models transition from research prototypes to validated decision-support tools. Integrating network science and topic-evolution modeling would further clarify how subfields, collaborations, and methodological innovations emerge over time.

Finally, future efforts should prioritize interdisciplinary and regulatory engagement, including participatory research with pharmaceutical developers, regulators, and methodologists. Establishing community standards for reporting, validation, documentation, and reproducibility, particularly for ML-augmented workflows, will help address the current barriers to regulatory acceptance. Collectively, these future directions can strengthen the methodological rigor, transparency, and practical impact of ML in advancing model-informed drug development.

## 6. Conclusions

This bibliometric and systematic review provides a concise overview of global trends in ML–MIDD research from 2015 to 2025, showing rapid growth in hybrid modeling strategies, expanding applications across the drug development pipeline, and increasing attention to regulatory readiness. While ML-driven tools, such as neural PBPK emulators, deep learning-assisted covariate inference, and NLP-enabled evidence synthesis, have enhanced pharmacometric efficiency, persistent challenges related to data quality, model transparency, and validation remain critical. Based on these findings, we recommend prioritizing (i) the development of accessible, high-quality shared datasets; (ii) standardized validation and reporting frameworks for ML-integrated pharmacometric models; and (iii) closer collaboration among pharmacometric, data science, and regulatory communities to ensure trustworthy and decision-grade implementation. These steps will support the continued evolution of ML-enabled MIDD toward a more reproducible, interpretable, and clinically impactful discipline.

## Figures and Tables

**Table 1:** Representative studies highlighting the integration of machine learning in model-informed drug development (ML-MIDD). This table presents a selection of the most relevant studies for ML-MIDD, with additional context linking bibliometric clusters to methodological approaches, sample sizes, validation, and performance metrics where available. The full list of curated studies is provided in Supplementary Data S5.

Author(s)	Year of Publication	Main Findings Related to ML-MIDD	Sample/Validation/Performance
Courlet P et al. [38]	2023	Integrated ML-selected radiomics covariates with EHR and imaging to predict tumor growth in melanoma. Identified key clinical/genomic factors.	91 patients; 311 tumor measurements; Radiomics subcohort 38 patients; ML covariates explained 75% TTB0 & 71% k growth IIV; cross-validation R^2^ = 0.72 ± 0.05 (TTB0), 0.36 ± 0.05 (k growth).
Ryeznik Y et al. [39]	2025	Pharmacometric-informed trial optimization framework using NLMEM; compared statistical strategies in rare neurological diseases.	Simulated *n* = 100 (50/arm), 128 scenarios × 2500 repetitions; performance: type I error, power, sensitivity metrics.
Franssen LC et al. [40]	2022	QSP-based Immunogenicity Simulator integrating bioinformatics, PK, and immune response; evaluated predictive credibility across stages.	10 mAbs, Phase I–III; subject-level PK/ADA data; % study duration with predictions within observed 95% CI.
Ribba B et al. [41]	2022	RL with PK-PD simulations for individualized propofol dosing; adaptive learning from simulated or retrospective patient data.	Virtual populations 100–500 patients; RMSE vs. standard dosing; model-enhanced RL reduced error by up to 90%.
Li X et al. [42]	2023	ML-PBPK platform to predict human PK without in vitro assays; improved predictions over traditional PBPK.	2292–6083 compounds for ML training; 40 test molecules; Best model R^2^ = 0.899–0.950; PBPK AUC within 2-fold: 65% vs. 47.5%.
Gomeni R et al. [43]	2025	ML-augmented propensity score framework to control placebo responses; improved treatment effect estimation.	Two RCTs: *n* = 459 & *n* = 512; ROC AUC 0.81–0.88; Sensitivity 0.75–0.83; Specificity 0.88–0.91.
Essenburg C et al. [44]	2025	Mixed-effects modeling to assess APOE4 impact on Alzheimer’s progression; integrated genetics, biomarkers, imaging.	2092 participants, 13,699 follow-ups; median 5 visits per subject; validated across CN, SMC, EMCI, LMCI, AD.
Wang Y et al. [45]	2019	ML/deep learning and radiomics support MIDD in dosing, trial design, regulatory decisions.	Various examples; validated via clinical outcomes, simulations, expert review; no single patient-level dataset.
Chan P et al. [46]	2022	ML-assisted MBMA for efficacy and safety in biologics and small molecules; expedited database building and missing data imputation.	Case 1: 102 articles, 21,305 patients; Case 2: RA database 201 studies, 64,471 patients; model evaluation via posterior predictive checks & internal validation.
Martins FS et al. [47]	2023	ANN-MLP + PBBM for sustained-release metformin optimization; confirmed bioequivalence.	13 training + 5 validation formulations; 10 virtual crossover trials, 36 subjects; R^2^ ≥ 0.99; GMR 90% CI within 80–125%.
Rohleff J et al. [48]	2025	VAE framework for NLME modeling; simultaneous population parameter estimation and covariate selection.	Theophylline PK: covariates selected in 1 run; Neonatal weight: 50 neonates, 1 run vs. 2–244 runs traditional; CPU 26% longer than single fit without covariate selection.
Nagpal S et al. [49]	2024	MIDD in nanomedicine integrating PBPK/PBNB, IVIVC, AI-assisted optimization; predicted in vivo performance and release specifications.	Validation: model-based with partial human data; in vitro/ex vivo data to inform simulations; Level A-IVIVC examples cited.

Note: IIV = inter-individual variability; TTB0/k growth = tumor baseline volume and growth rate parameters in tumor growth inhibition models; ROC AUC = area under the receiver operating characteristic curve; GMR 90% CI = geometric mean ratio with 90% confidence interval for bioequivalence; CN/SMC/EMCI/LMCI/AD = cognitively normal/subjective memory complaint/early mild cognitive impairment/late mild cognitive impairment/Alzheimer’s disease; R^2^ = coefficient of determination indicating predictive performance; PBPK/PBBM/IVIVC = physiologically based pharmacokinetics/physiologically based biopharmaceutics modeling/in vitro–in vivo correlation; VAE/NLME = variational autoencoder/nonlinear mixed-effects modeling.

## Data Availability

The original contributions presented in this study are included in the article/Supplementary Material. Further inquiries can be directed to the corresponding author.

## Supplementary Materials

The following supporting information can be downloaded at: https://www.mdpi.com/article/10.3390/pharmaceutics18050542/s1, Supplementary Data S1: Full Search Strategy; Supplementary Data S2: Pseudocode Representing the Custom R Workflow for ML-MIDD Analysis; Supplementary Data S3: Research Questions, Descriptions, and Analytical Strategies; Supplementary Data S4: PRISMA Checklist; Supplementary Data S5: Bibliometric Analysis Supplemental Results; Supplementary Data S6: Curated Dataset Used for Systematic Review Analysis.

## Abbreviations

The following abbreviations are used in this manuscript:

AI	Artificial Intelligence
ANN	Artificial Neural Network
CAGR	Compound Annual Growth Rates
CSV	Comma-Separated Values
D-MPNN	Deep Message Passing Neural Network
DL	Deep Learning
DOI	Digital Object Identifier
EHR	Electronic Health Record
IVIVC	In Vitro-In Vivo Correlation
LIME	Local Interpretable Model-agnostic Explanations
LLM	Large Language Model
MBMA	Model-Based Meta-Analysis
MCP	Multiple-Country Publications
MeSH	Medical Subject Headings
MIDD	Model-Informed Drug Development
ML	Machine Learning
MLP	Multilayer Perceptron
NLME	Non-Linear Mixed-Effects
PBBM	Physiologically Based Biopharmaceutics Modeling
PBPK	Physiologically Based Pharmacokinetic
PK/PD	Pharmacokinetic/Pharmacodynamic
PopPK	Population Pharmacokinetic
PRISMA	Preferred Reporting Items for Systematic Reviews and Meta-Analyses
QbD	Quality by Design
QSP	Quantitative Systems Pharmacology
RIS	Research Information Systems
RQ	Research Question
RWD	Real-World Data
SAEM	Stochastic Approximation Expectation-Maximization
SCM	Stepwise Covariate Modeling
SCP	Single-Country Publications
SHAP	SHapley Additive exPlanations
VAE	Variational Autoencoder
VBE	Virtual Bioequivalence
XAI	Explainable Artificial Intelligence
