# Supplementary material for: Global Trends in Integrating Machine Learning (ML) with Model-Informed Drug Development (MIDD): A Bibliometric and Systematic Review (2015–2025)
Source: Pharmaceutics. 2026 Apr 28;18(5):542. doi: 10.3390/pharmaceutics18050542 (PMC13210285; doi:10.3390/pharmaceutics18050542)
Supplement: Supplementary file 1 [file pharmaceutics-18-00542-s001.zip › Supplementary Data S1 - Full Search Strategy.pdf]

Supplementary Data S1

Full Search Strategy

A comprehensive and systematic search strategy was developed to identify peer-reviewed publications applying or integrating machine learning (ML), artificial intelligence (AI), or related computational methods within the framework of model-informed drug development (MIDD). The search strategy followed the Preferred Reporting Items for Systematic Reviews and Meta-Analyses extension for Scoping Reviews (PRISMA-ScR) to ensure transparency, reproducibility, and methodological rigor.

Three core conceptual domains guided the development of search terms:

1. Machine Learning and Artificial Intelligence

- Keywords: “machine learning,” “artificial intelligence,” “deep learning,” “neural network,” “AI/ML”

2. Model-Informed Drug Development (MIDD) and Pharmacometric Modeling

- Keywords: “model-informed drug development,” “MIDD,”
  “physiologically based pharmacokinetic,” “PBPK,”
  “quantitative systems pharmacology,” “QSP,”
  “population pharmacokinetics,” “PopPK,”
  “pharmacokinetic modeling,” “pharmacometrics,” “dose optimization”

3. Drug Development and Precision Therapeutics Context

- Keywords: “drug development,” “precision pharmacology,” “predictive modeling,” “dose selection”

Where applicable, controlled vocabulary terms, particularly Medical Subject Headings (MeSH) in PubMed, were incorporated to increase retrieval precision. Boolean operators (AND/OR), phrase searching, and database-specific field tags (e.g., TS= in Web of Science) ensured optimal performance and reproducibility across platforms.

The search was restricted to January 1, 2015 to September 30, 2025, aligning with the period of rapid expansion in ML/AI methodologies and their integration into PBPK, QSP, and broader MIDD workflows.

All retrieved records were exported in standardized bibliographic formats (BibTeX, RIS, CSV) for downstream harmonization, deduplication, and bibliometric processing.

Database	Query	Results
Web of Science	TS=(("machine learning" OR "artificial intelligence" OR "deep learning" OR "neural network") AND ("model-informed drug development" OR "MIDD" OR "PBPK" OR "physiologically based pharmacokinetic" OR "QSP" OR "quantitative systems pharmacology" OR "PopPK" OR "population pharmacokinetics" OR "pharmacokinetic modeling" OR "dose optimization" OR "pharmacometrics")) AND PY=(2015-2025) AND LA=(English)	322

SCOPUS	("machine learning" OR "artificial intelligence" OR "deep learning" OR "neural network") AND ("model-informed drug development" OR "PBPK" OR "QSP" OR "PopPK" OR "pharmacokinetic modeling" OR "dose optimization") AND ("drug development" OR "pharmacometrics" OR "precision pharmacology") AND PUBYEAR > 2014 AND PUBYEAR < 2026 AND (LIMIT-TO (DOCTYPE , "ar")) AND (LIMIT-TO (LANGUAGE , "English")) AND (LIMIT-TO (PUBSTAGE , "final")) AND (LIMIT-TO (EXACTKEYWORD , "Machine Learning"))	343
PubMed	("machine learning" OR "artificial intelligence" OR "deep learning" OR "neural network") AND ("model-informed drug development" OR "PBPK" OR "QSP" OR "PopPK" OR "pharmacokinetic modeling" OR "dose optimization") AND ("drug development" OR "pharmacometrics" OR "precision pharmacology")	105

Databases and Sources

To ensure comprehensive coverage of research integrating ML with MIDD, the literature search was conducted across three major bibliographic databases:

- Web of Science Core Collection: for multidisciplinary scientific literature and detailed citation metadata.
- Scopus: for broad coverage of pharmaceutical sciences, modeling research, and computational methodologies.
- PubMed/MEDLINE: for biomedical and clinical pharmacology research and for its controlled vocabulary indexing (MeSH).

These databases were selected for their complementary indexing scopes and their strengths in supporting bibliometric, citation, and network analyses.

Inclusion and Exclusion Criteria

Inclusion Criteria

Studies were considered eligible if they met all of the following criteria:

- Publication period: January 2015 to September 2025.
- Topical focus: Articles explicitly applying, integrating, or developing ML, AI, or deep learning approaches within MIDD contexts (e.g., PBPK, PopPK, QSP, exposure–response modeling, dose optimization).
- Research type: Original research presenting methodological advancements, model development, or applied ML–MIDD analyses.
- Domain relevance: Direct relevance to drug discovery, drug development, or clinical pharmacology.
- Language: Published in English.

Exclusion Criteria

Studies were excluded if they:

- Lacked sufficient bibliographic metadata for bibliometric processing.
- Focused on AI/ML applications unrelated to pharmacometrics or MIDD, such as imaging, diagnostics, or general EHR prediction without pharmacokinetic/pharmacodynamic relevance.
- Addressed non-pharmacometric modeling, including purely biological, computational, or engineering tasks without drug development implications.
- Were editorials, commentaries, perspectives, or theoretical works without new model development or original data.
- Were not published in English or lacked accessible titles/abstracts.

This framework ensured that the final dataset represented rigorous, original contributions central to the evolution of ML-enabled pharmacometric and MIDD methodologies.

Search Period and Updates

The search covered January 1, 2015 through September 30, 2025, capturing the decade during which ML and AI became increasingly integrated into PBPK, QSP, PopPK, and broader MIDD workflows. To ensure completeness, the search will be rerun immediately prior to final manuscript submission to incorporate newly published literature.

Transparency and Reproducibility

To enhance methodological transparency and facilitate independent verification, the complete database-specific search strings, filters, and field tags are provided in Supplementary Data 1. All retrieved records were exported in standardized formats (BibTeX, RIS, CSV), and the curated dataset used for scoping review analysis is provided in Supplementary Data 6.
