# Supplementary material for: Global Trends in Integrating Machine Learning (ML) with Model-Informed Drug Development (MIDD): A Bibliometric and Systematic Review (2015–2025)
Source: Pharmaceutics. 2026 Apr 28;18(5):542. doi: 10.3390/pharmaceutics18050542 (PMC13210285; doi:10.3390/pharmaceutics18050542)
Supplement: Supplementary file 1 [file pharmaceutics-18-00542-s001.zip › Supplementary Data S2 - Pseudocode Representing the Custom R Workflow for ML-MIDD Analysis.pdf]

Supplementary Data S2

Pseudocode Representing the Custom R Workflow for ML-MIDD Analysis

1. Set Working Directory
   - Define project folder path for all files and outputs

2. Load Required Libraries
   - bibliometrix, openxlsx
   - ggplot2, cowplot, grid, stringi, VennDiagram
   - Install any missing packages

3. Read Bibliographic Data from Sources
   - Scopus (.bib)
   - Web of Science (.bib)
   - PubMed (.txt)
   - Convert each dataset to a standardized data frame

4. Merge Datasets and Remove Duplicates
   - Merge all three sources
   - Remove duplicate records based on DOI, title, author, and year
   - Export merged dataset to Excel, CSV, and TXT formats

5. Generate Normalized Unique IDs
   - Normalize DOI, title, author names
   - Create composite IDs using first author, year, and title
   - Assign unique IDs to each record for all sources

6. Compute Intersection Counts Across Databases
   - Determine records unique to each source
   - Determine overlaps between pairs and all three sources
   - Align counts with the deduplicated merged dataset
   - Adjust counts if necessary to ensure perfect alignment

7. Generate Venn Diagram for Publication Overlap
   - Create triple Venn diagram with counts from step 6
   - Customize colors, labels, and styles for publication-ready figure
   - Convert Venn diagram to a ggplot-compatible object

8. Generate Bar Chart of Publications per Database
   - Create a data frame with counts for Web of Science, Scopus, and PubMed
   - Plot bar chart with counts and customized aesthetics

9. Combine Venn Diagram and Bar Chart
   - Stack figures vertically using plot grid
   - Add title reflecting total deduplicated records
   - Save final figure as high-resolution PNG

10. Optional Interactive Exploration
   - Launch Biblioshiny for further bibliometric analysis
