# Supplementary material for: Global Trends in Integrating Machine Learning (ML) with Model-Informed Drug Development (MIDD): A Bibliometric and Systematic Review (2015–2025)
Source: Pharmaceutics. 2026 Apr 28;18(5):542. doi: 10.3390/pharmaceutics18050542 (PMC13210285; doi:10.3390/pharmaceutics18050542)
Supplement: Supplementary file 1 [file pharmaceutics-18-00542-s001.zip › Supplementary Data S3 - Research Questions.pdf]

Supplementary Data S3

Research Questions, Descriptions, and Analytical Strategies Used in the Bibliometric Analysis

No.	Research Question (RQ)	Description	Analysis / Strategy to Answer
1	What is the current state of publication?	This question deals with the current form or the basic information of the dataset, such as the document type, source type, subject area, and language.	1. Document type<br>2. Source type<br>3. Subject area<br>4. Language<br>5. Total number of contributors, i.e., total number of authors, institutions, countries, or source titles that contribute to the publication.
2	What is the current publication trend?	Present the number of publications by year, either using a table or chart. Information to be presented includes the publication count, total citations, h-index, g-index, and m-index.	1. Number of publications by year<br>2. Annual growth rate<br>3. Evolution of publications, i.e., based on the history, phases, or development of the literature.
3	What are the most productive contributors?	The publication's contributors include the authors, institutions, countries, and source titles. Productive contributors can be measured by the number or quantity (or productivity) of publications produced to date.	1. Most productive authors<br>2. Most productive institutions<br>3. Most productive countries<br>4. Most productive source titles (or journals)<br>5. Bradford's law
4	What are the most influential contributors?	Influential contributors can be measured by the number of citations they received, total h-index, g-index, or m-index.	1. Most influential authors<br>2. Author's production over time<br>3. Most influential institutions<br>4. Most influential countries<br>5. Most influential source titles (or journals)
5	What are the authorship patterns of the publication?	This analysis focuses on the authorship patterns as well as the performance of publications based on analysis related to the authorship.	1. Total number of contributing authors per document<br>2. Productivity patterns of authors and research contributions<br>3. Lotka's law

No.	Research Question (RQ)	Description	Analysis / Strategy to Answer
**6**	What is the current state of collaboration?	This question is also related to the authorship of the publication but looks further into the collaboration of authors. It is also related to the social structure of the publication. Based on author affiliations, collaboration among institutions and countries can be generated.	1. Single-authored vs. multi-authored documents<br>2. Documents per author<br>3. Co-authors per document<br>4. Collaboration by authors<br>5. Collaboration by institutions<br>6. Collaboration by countries<br>7. Collaboration index
**7**	Which are the most influential articles (or documents)?	This analysis will measure the documents based on the number of citations they have received so far.	1. Highly cited documents
**8**	What is the intellectual structure of current research?	This question is associated with the citation analysis, which shows how publications related to specific research domains influence the scientific community.	1. Citation metrics, including information about citations per paper, citations per author, total h-index, g-index, m-index<br>2. Most local cited documents<br>3. Most global cited documents<br>4. Most local cited references<br>5. References spectroscopy<br>6. Bibliographical coupling<br>7. Co-citation analysis<br>8. Historiography
**9**	Which themes are the most popular among scholars?	The theme analysis (also called clustering analysis) is based on the keywords, title, or abstract of the publication. This analysis can also present the overall thematic structure of the literature.	1. Co-occurrence networks<br>2. Thematic map<br>3. Most frequent keywords<br>4. Word cloud analysis<br>5. Tree map
**10**	How has the theme of the publication evolved?	To answer this question, you must present the keywords, words, or themes that have evolved over time. The output from this analysis can also help predict future research directions in the field.	1. Thematic evolution<br>2. Trend topic<br>3. Word dynamic
