# Supplementary material for: Global Trends in Integrating Machine Learning (ML) with Model-Informed Drug Development (MIDD): A Bibliometric and Systematic Review (2015–2025)
Source: Pharmaceutics. 2026 Apr 28;18(5):542. doi: 10.3390/pharmaceutics18050542 (PMC13210285; doi:10.3390/pharmaceutics18050542)
Supplement: Supplementary file 1 [file pharmaceutics-18-00542-s001.zip › Supplementary Data S5 - Bibliometric Supplemental.pdf]

Supplementary Data S5

Bibliometric Analysis Supplemental Results

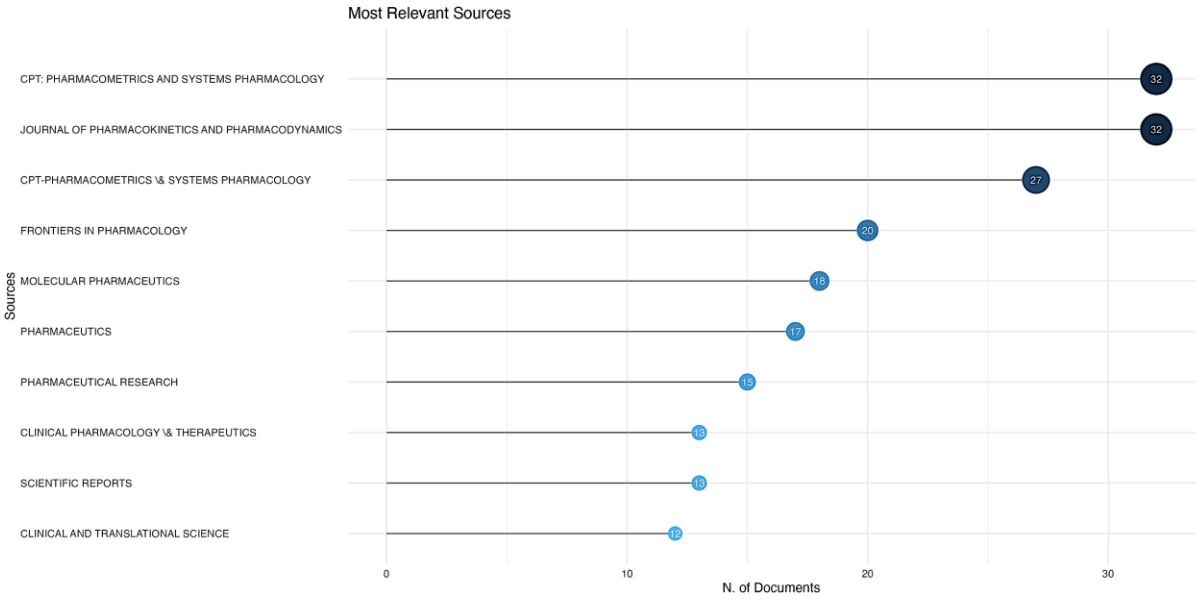

FIGURE S1
Most Relevant Sources.

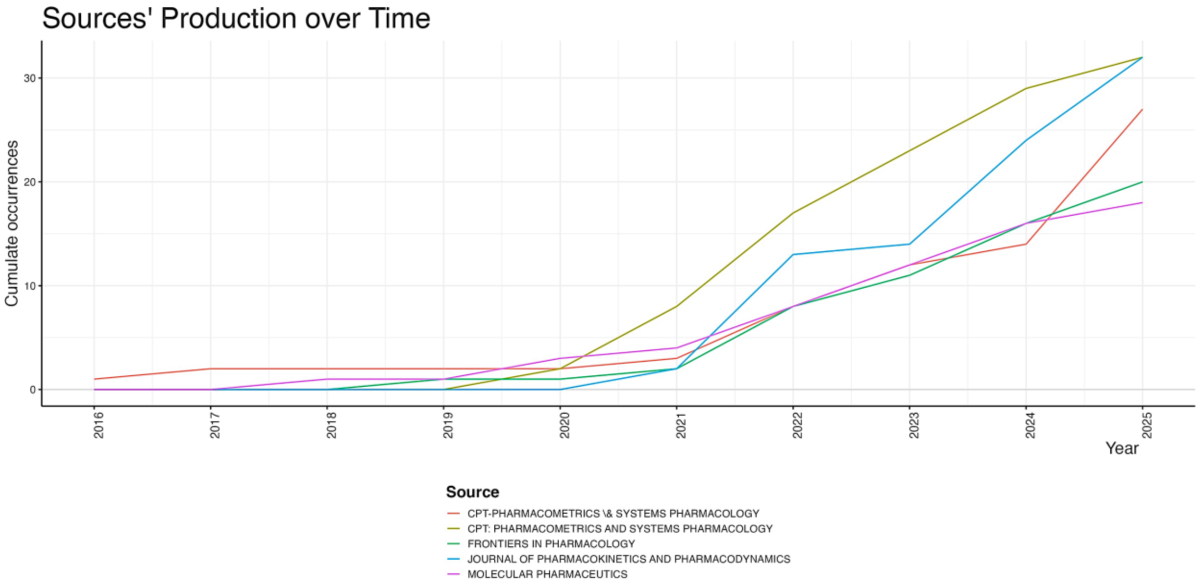

FIGURE S2
Sources' Production over Time.

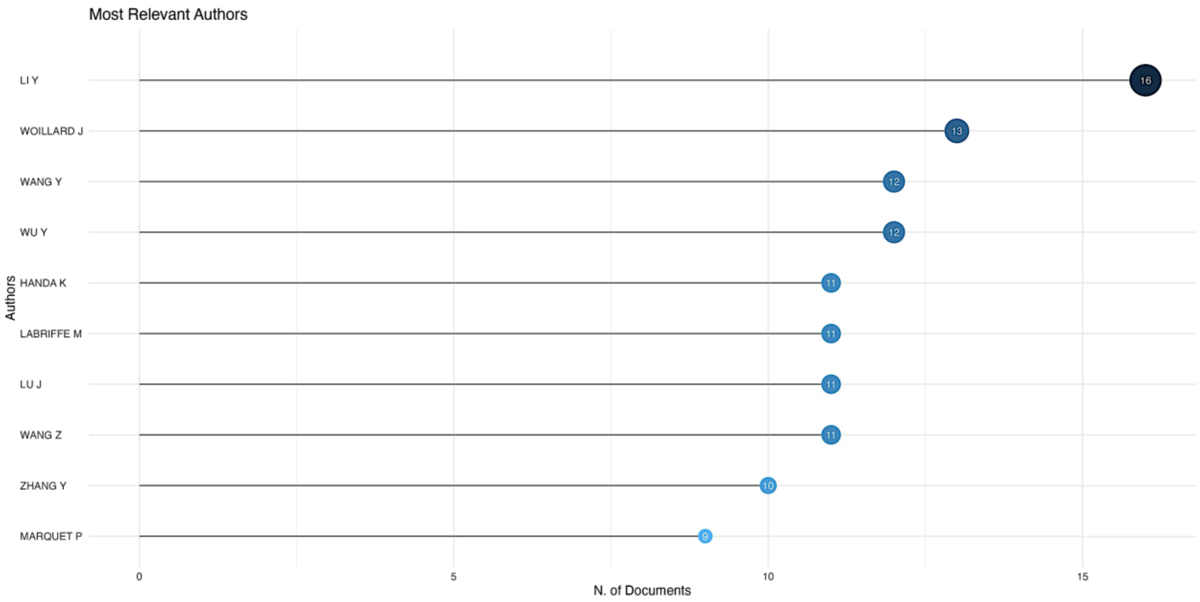

FIGURE S3

Most Relevant Authors.

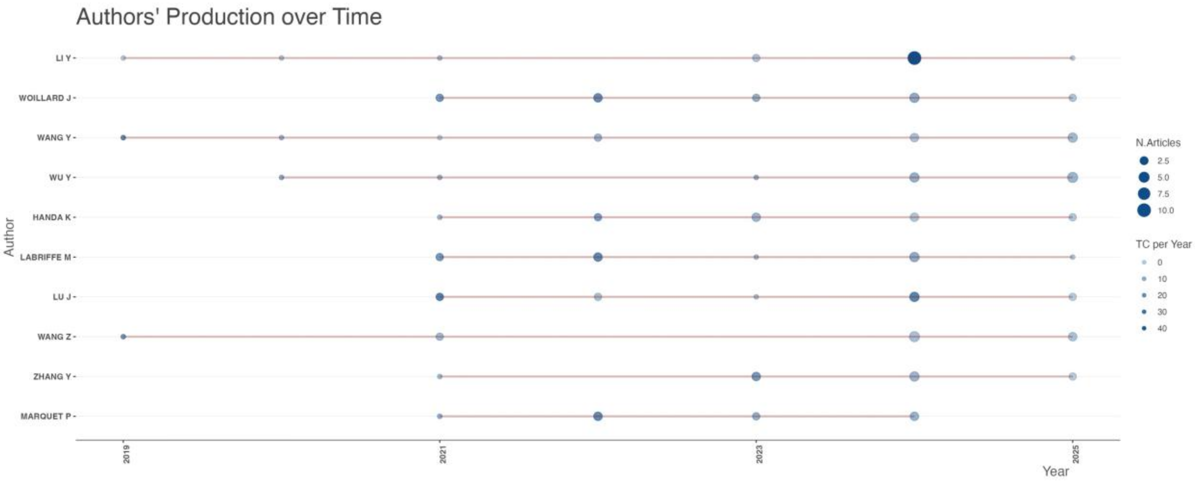

FIGURE S4

Authors' Production over Time.

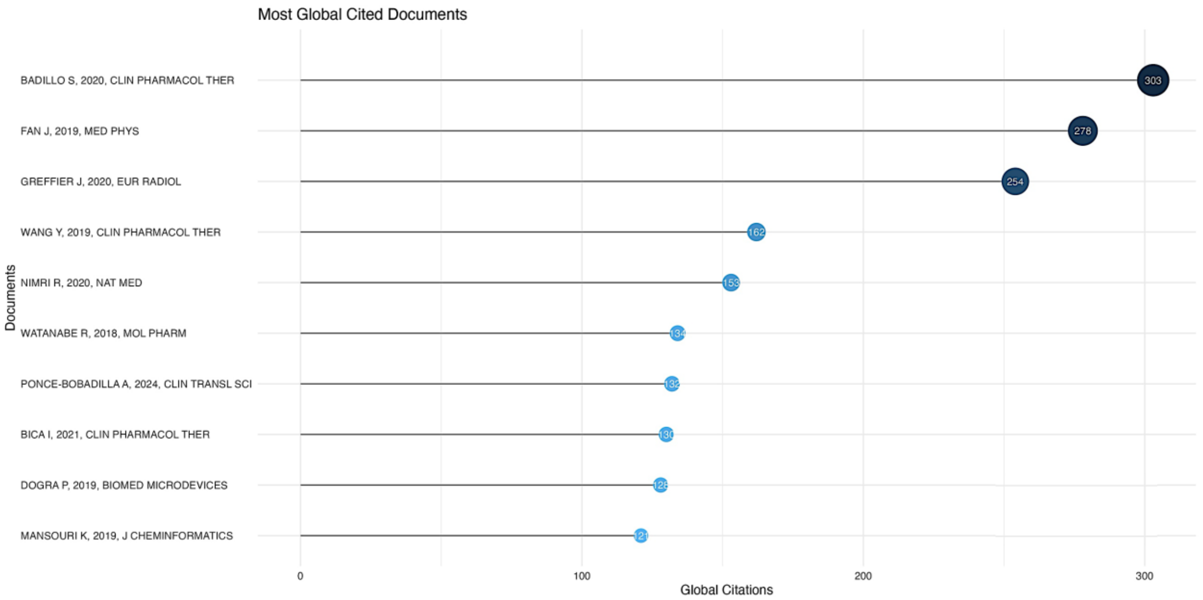

FIGURE S5

Most Global Cited Documents.

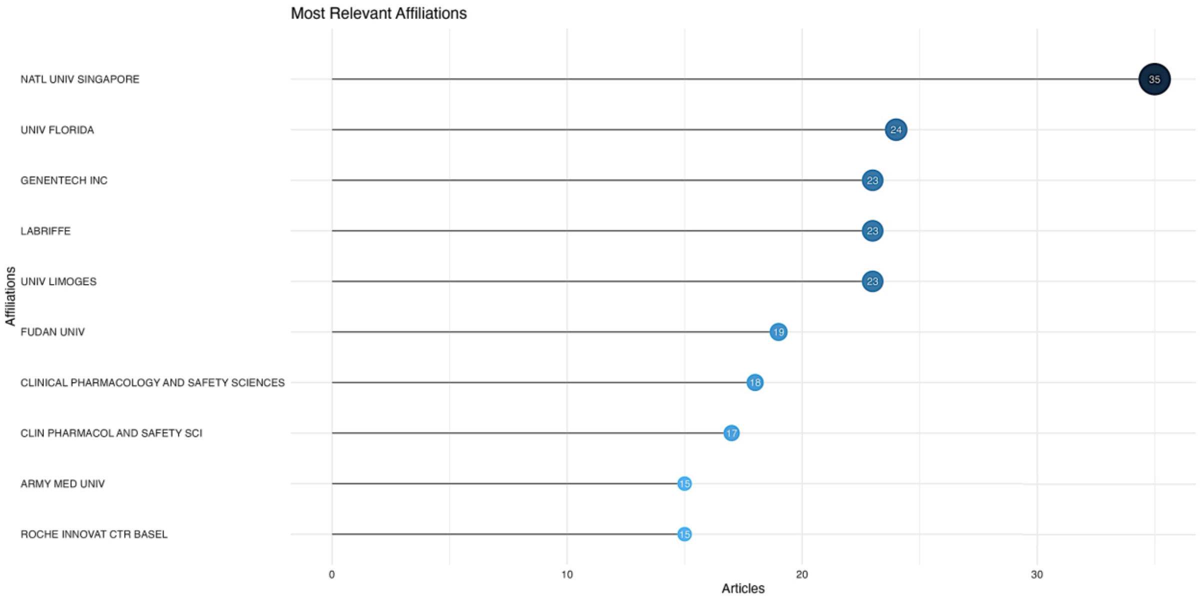

FIGURE S6

Most Relevant Affiliations.

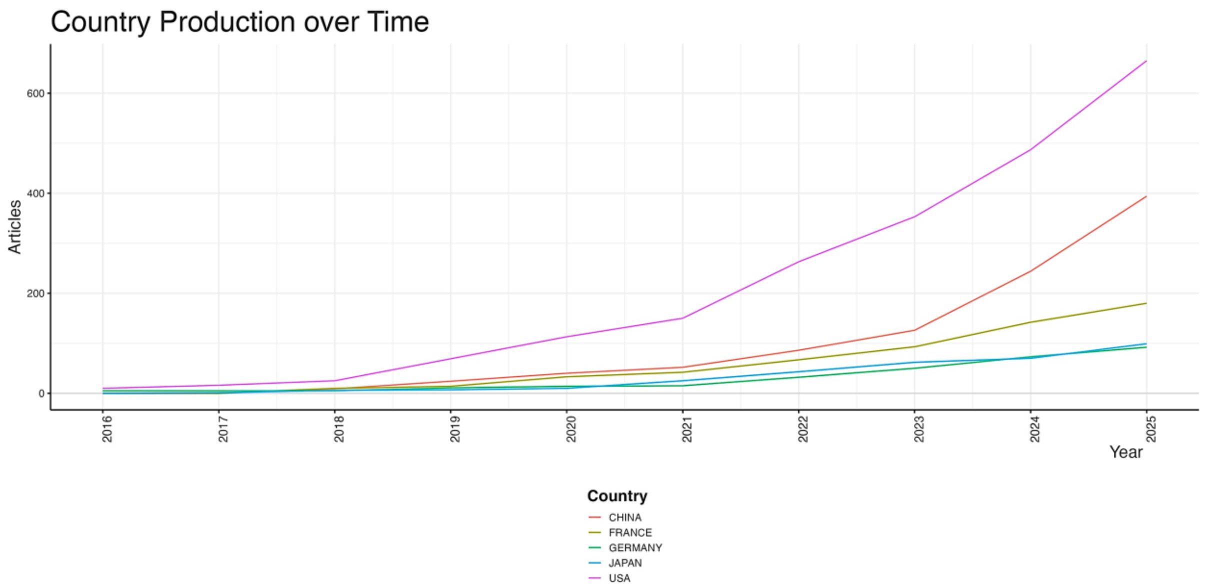

FIGURE S7

Country Production over Time.

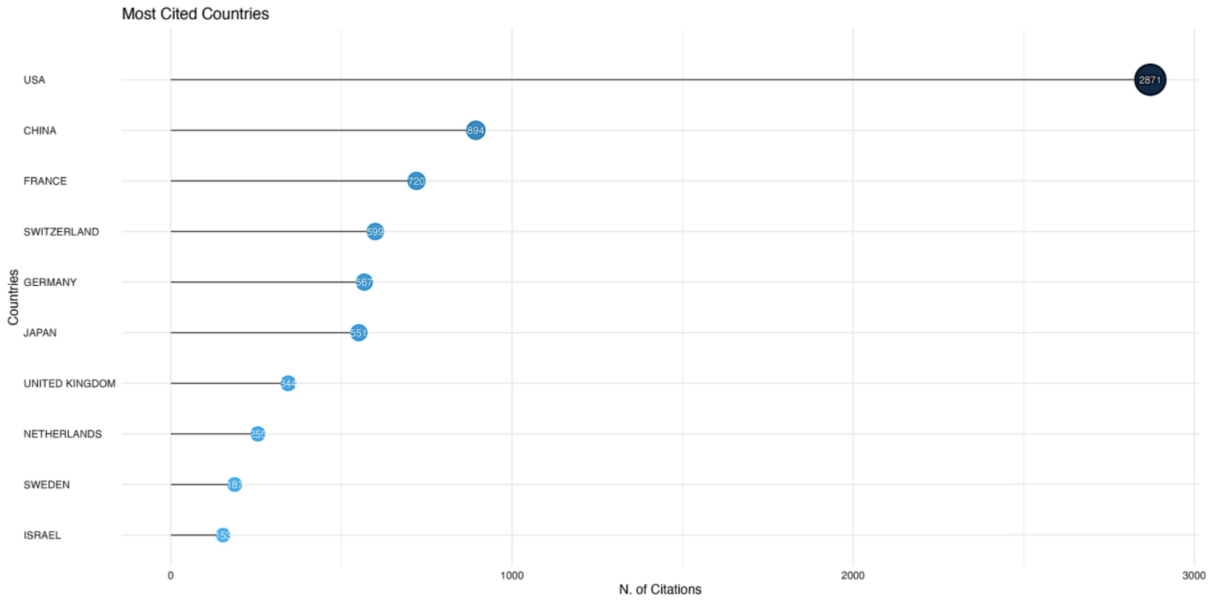

FIGURE S8

Most Cited Countries.

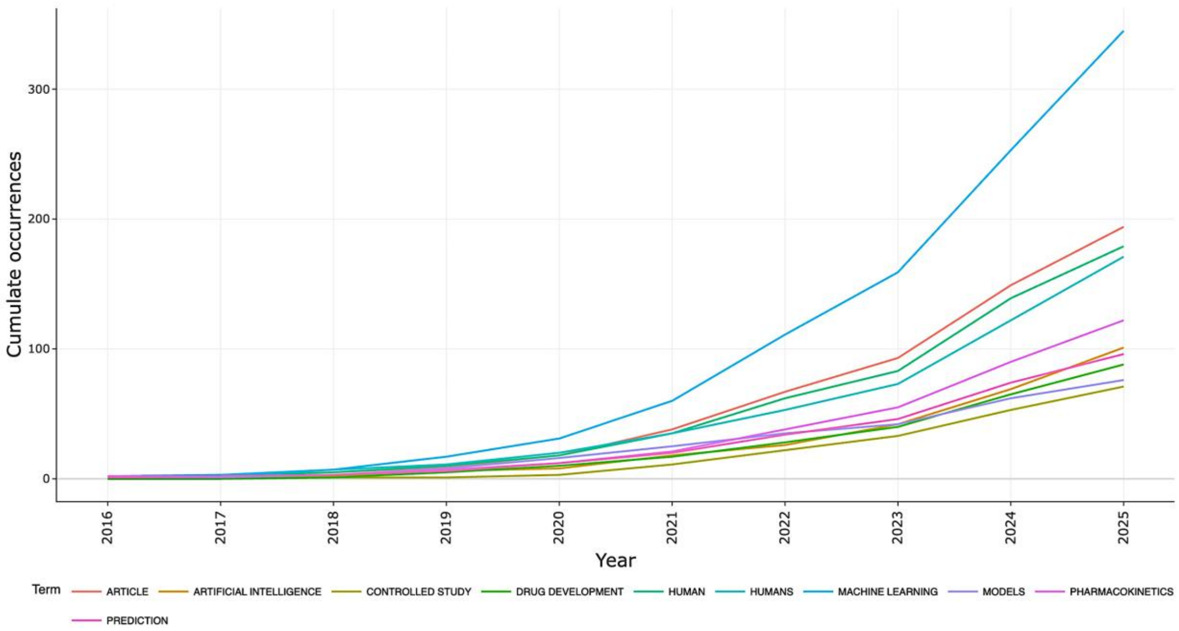

FIGURE S9

Words' Frequency over Time.
